# Molecular hydrogen promotes wound healing by inducing early epidermal stem cell proliferation and extracellular matrix deposition

**DOI:** 10.1186/s41232-023-00271-9

**Published:** 2023-03-28

**Authors:** Pengxiang Zhao, Zheng Dang, Mengyu Liu, Dazhi Guo, Ruiliu Luo, Mingzi Zhang, Fei Xie, Xujuan Zhang, Youbin Wang, Shuyi Pan, Xuemei Ma

**Affiliations:** 1 Faculty of Environment and Life, Beijing University of Technology, Beijing, 100124 People’s Republic of China; 2 Beijing Molecular Hydrogen Research Center, Beijing, 100124 People’s Republic of China; 3 Beijing International Science and Technology Cooperation Base of Antivirus Drug, Beijing, 100124 People’s Republic of China; 4 Department of Hyperbaric Oxygen, Sixth Medical Center of PLA General Hospital, Beijing, 100048 People’s Republic of China; 5 Department of Plastic Surgery, Peking Union Medical College Hospital (Dongdan campus), No. 1 Shuaifuyuan Wangfujing Dongcheng District, Beijing, 100730 People’s Republic of China

**Keywords:** Molecular hydrogen, Wound care, Extracellular matrix deposition, Epidermal stem cell proliferation, Re-epithelialization

## Abstract

**Background:**

Despite progress in developing wound care strategies, there is currently no treatment that promotes the self-tissue repair capabilities. H_2_ has been shown to effectively protect cells and tissues from oxidative and inflammatory damage. While comprehensive effects and how H_2_ functions in wound healing remains unknown, especially for the link between H_2_ and extracellular matrix (ECM) deposition and epidermal stem cells (EpSCs) activation.

**Methods:**

Here, we established a cutaneous aseptic wound model and applied a high concentration of H_2_ (66% H_2_) in a treatment chamber. Molecular mechanisms and the effects of healing were evaluated by gene functional enrichment analysis, digital spatial profiler analysis, blood perfusion/oxygen detection assay, in vitro tube formation assay, enzyme-linked immunosorbent assay, immunofluorescent staining, non-targeted metabonomic analysis, flow cytometry, transmission electron microscope, and live-cell imaging.

**Results:**

We revealed that a high concentration of H_2_ (66% H_2_) greatly increased the healing rate (3 times higher than the control group) on day 11 post-wounding. The effect was not dependent on O_2_ or anti-reactive oxygen species functions. Histological and cellular experiments proved the fast re-epithelialization in the H_2_ group. ECM components early (3 days post-wounding) deposition were found in the H_2_ group of the proximal wound, especially for the dermal col-I, epidermal col-III, and dermis-epidermis-junction col-XVII. H_2_ accelerated early autologous EpSCs proliferation (1–2 days in advance) and then differentiation into myoepithelial cells. These epidermal myoepithelial cells could further contribute to ECM deposition. Other beneficial outcomes include sustained moist healing, greater vascularization, less T-helper-1 and T-helper-17 cell-related systemic inflammation, and better tissue remodelling.

**Conclusion:**

We have discovered a novel pattern of wound healing induced by molecular hydrogen treatment. This is the first time to reveal the direct link between H_2_ and ECM deposition and EpSCs activation. These H_2_-induced multiple advantages in healing may be related to the enhancement of cell viability in various cells and the maintenance of mitochondrial functions at a basic level in the biological processes of life.

**Supplementary Information:**

The online version contains supplementary material available at 10.1186/s41232-023-00271-9.

## Introduction

Wound healing in adult mammals generally involves four major processes [1–4]: haemostasis, inflammation, proliferation, and remodelling—which may leave a scar [5, 6]. Failure at any of these stages could lead to acute or chronic wound repair disorders [7]. In evaluating wound healing, the acronym “TIME” (Tissue, Infection/Inflammation, Moisture balance, and Edge of wound) has been used to describe a favourable wound-bed microenvironment [8, 9]. Throughout the dynamic healing process, rapid re-epithelialization is mediated by epidermal stem cells (EpSCs) and the extracellular matrix (ECM) to restore the skin barrier [10–12]. EPSCs can be activated and recruited from various tissues [13], such as hair follicles (HFs), interfollicular epidermis (IFE), and bone marrow [14–17], thus replenishing the keratinocytes at the wound site and facilitating wound closure [18, 19]. Furthermore, ECM molecules can stimulate keratinocyte migration from the edges of the wound and nearby tissues to the wound bed [20, 21]. In addition to serving as a scaffold [22], the ECM is instrumental in creating a favourable environment for skin progenitor cells [23]. This is largely due to collagen in the ECM [24], which is synthesized by fibroblasts, epithelial cells [25], and keratinocytes [12] , and constitutes a basis for translational wound-repair medications and therapies [26–29]. However, the process of healing is multifaceted, and improvements in wound healing, including safer and more efficient wound-healing methods, are needed.

Wound healing processes involve the spatial and temporal synchronization of a variety of cell types with distinct roles in all phases [30]. During these, the reactive oxygen species (ROS) gradient is considered as one of the first signals that activate the cellular response and also crucial to regulate several other phases of healing processes [31]. While key factors that promote wound healing also include avoiding the tissue damage caused by the overproduction of ROS and activating the functions of tissue repair.

Hydrogen gas has been reported by Ohsawa et al. [32] in 2007 to be an efficient antioxidant in protecting rats brain from ischemia-reperfusion injury. Other studies added evidence of H_2_ therapy in massive oxidative damage and inflammatory diseases [33–36]. Latest researches suggest that hydrogen can also be beneficial on skin injury, for example, H_2_ has been shown to effectively improve the damage repair of cutaneous wound [37], burn wound [38], pressure ulcer [39, 40], diabetic wounds [41, 42], radiation injury [43], psoriasis damage [44], and oral-wound [45]. Current explanations for hydrogen promoting wound healing have been mostly focused on its ability of anti-inflammatory, anti-oxidative stress and reacting with cytotoxic ROS. Few studies indicated that H_2_ may function in stem cell viability and collagen synthesis. Kawasaki et al. [46] suggested hydrogen gas prolonged replicative lifespan of bone marrow cells in vitro, and Zhang et al. [47] pointed out that hydrogen protected hematopoietic stem cell from radiation injury by reducing hydroxyl radical. In a study of pressure ulcer, masson staining was used to prove hydrogen inhalation induced collagen synthesis [40], another study of diabetic wound, topical application of H_2_ was revealed to induce Col-1 [42]. More comprehensive and deep research of hydrogen on wound healing are still needed, and many questions remains to be answered, such as how H_2_ functions on different types of cells especially on stem cells activities? What types of collagens and other ECM are synthesized under H_2_ treatment? Is this hydrogen-promoted wound healing dose-dependent?

In the present study, by using a full-thickness dorsal-skin defect mouse model, we revealed that daily treatment in an high concentration H_2_ (66% H_2_ + 33% O_2_) chamber induced a novel pattern of wound healing with less inflammation and better angiogenesis, faster cell migration, mitochondrial function maintenance, and less scab formation. H_2_ treatment facilitated early ECM deposition and EpSCs activation, thus providing a simple and effective approach to improve wound healing (mainly due to the high concentration of H_2_). H_2_-releasing dressing received a relevant effect in cutaneous wound healing. Therefore, H_2_ treatment may potentially provide a novel strategy different from the traditional pattern of wound care, with a vast range of applications in clinical postoperative treatment.

## Methods

### Cell culture

Human embryonic skin fibroblasts CCC-ESF-1 (ESF) (generation 10, G10), human embryonic lung fibroblast CCC-HPF-1 (HPF) (G10), human immortal keratinocyte line (HaCaT) (G8), and human Umbilical Vein Endothelial Cells (HUVECs) (G3) were obtained from the National Infrastructure of Cell Line Resource Center, Beijing, China. Primary mesenchymal stem cells derived from newborn umbilical cords (Human Umbilical Cord Primary Mesenchymal Stem Cells (HUCP-MSCs)) were donated by Beijing Obstetrics and Gynecology Hospital. Cells were routinely cultured at 5% CO_2_ and 37 °C in DMEM medium (for ESF, HPF, HaCaT, and HUVEC) (Gibco, NY, USA) supplemented with 10% fetal calf serum (Gibco, NY, USA) and 1% penicillin and streptomycin (Gibco, NY, USA).

### Animal experiments

All animal studies were carried out according to protocols approved by the Committee on Ethics of Biomedicine Research, the Sixth Medical Center, PLAGH, China, and all procedures were conducted in accordance with the Regulations for the Administration of Affairs Concerning Experimental Animals (China). Male C57BL/6J mice (7 weeks old, 20–24 g) were purchased from Vital River Laboratory Animal Technology Co., Ltd. (Beijing, China). Animals were maintained under standard conditions at 22 °C to 25 °C with a 12-h light/dark cycle and were fed a normal diet. Oral enrofloxacin (0.17 mg/mL) intake was administered in daily drinking water, and the antibiotic was prepared with chlorinate-free and acid-free water. For the NAC treatment group, 3.3 mg NAC (the ROS scavenger acetylcysteine; #S1623, Selleckchem, China) in 260 μL distilled water was injected in each mouse with 22 (± 2) g of body weight.

### Full-thickness cutaneous wound healing model

**Fig. 1:**
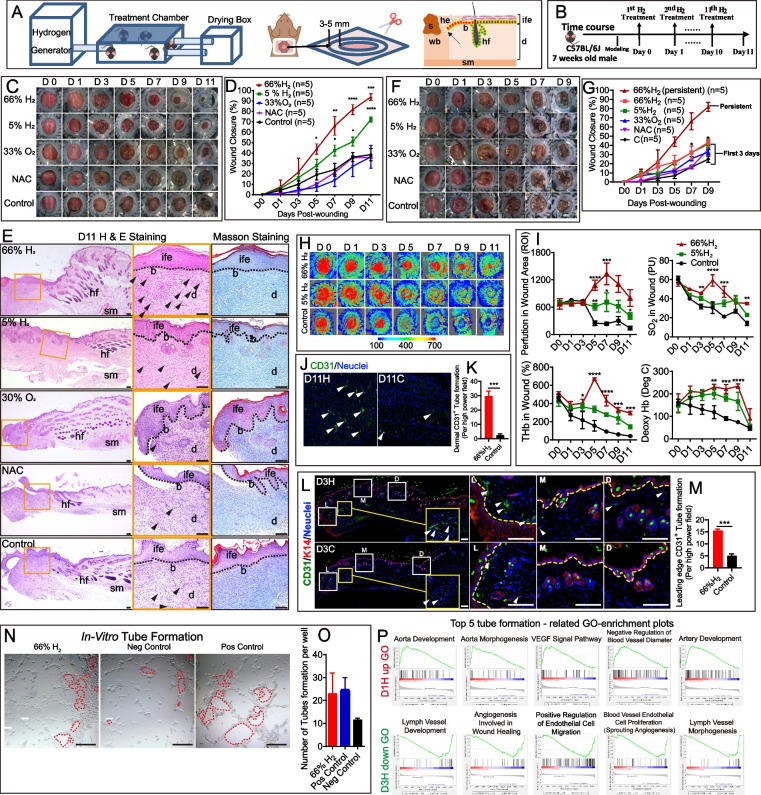
Persistent high concentration of H_2_ significantly accelerated cutaneous wound healing, blood perfusion and vessel formation. **A** Left: connection between each module in the H_2_ chamber system. Middle: Model of the full-thickness cutaneous wound in mouse backs; inside circle = original wound, outside circle = sampling boundary. Right: Scheme of a wound section after injury. **B** Timeline of animal experiments and daily H_2_ treatment (or under other conditions). Mice underwent surgery for 30 min and then immediately put in H_2_ chamber for 1 h. Thereafter, they were treated with H_2_ (or other conditions) every 24 h for 1 h until sacrifice. **C** Capture of wound area every two days after modeling in different persistent conditions (*n* = 5 for each group). **D** Comparison of wound closure percentage after modeling (persistent daily treatments). **E** H&E staining and Masson staining showing the tissue remodeling 11 days post-wounding (persistent daily treatments). **F** Treatment was applied for the first 3 days only, and the images of wound areas were captured every 2 days after modeling (days 0–9). **G** Comparison of wound closure percentage after modeling among the five groups (treatment for the first 3 days). **H** Blood flow perfusion 0–11 days post-wounding in the 66% H_2_, 5% H_2_, and Control groups. **I** Quantification of blood perfusion, SO_2_, tHb, and HHb in wound area among the three groups. **J**, **K** Representative immunofluorescence images and quantification for CD31^+^ (green) tube formation in dermal wounds of at day 11 post-wounding. **L**, **M** Panoramic scanning and the quantification of wound edge tube formation at the leading edge (L, 0–1 mm from wound site), mid-end (M, 1–2 mm from wound site) and distal (D, 2–3 mm from wound site) areas of the wound at day 3 post-wounding. Yellow dotted line indicates the boundary between the epithelium and dermis. **N**, **O** Representative microscopic images and the quantification of in vitro blood vessel formation of Human Umbilical Vein Endothelial Cells (HUVEC) at 12 h after different treatments. Red hatched line outlines the newly formed tubes. **P** GSEA Top-5 tube formation-related GO-bp enrichment plots showed the H_2_ accelerated tube formation (2 days earlier than the controls). Data in **D**, **G**, and **I** were analyzed by two-way ANOVA test, and data in **K**, **M**, and **O** were processed unpaired *t* test. All of the data are plotted as Mean ± SEM. *P value < 0.05; **P value < 0.01; ***P value < 0.001; no stars for *P* value > 0.05; *in **D** indicates a significant difference between the 66% H_2_ and control group, the 5% H_2_ and control group, separately; * in **G** indicates a significant difference between the 66% H_2_ and control group. Scale bar = 100 μm. Black dotted line in **E** indicates the boundary between the epithelium and dermis. Arrow in **A** (right) indicates the epithelial tongue; black arrowheads in **E** indicate blood capillaries in the dermal layer of the wound. b, basal layer; d, dermis; he, hypertrophic epidermal wound edge; hf, hair follicles; ife, interfollicular epithelium; s, scab; sm, smooth muscle; wb, wound bed

The full-thickness cutaneous wound healing model was established according to the Murine Model of Wound Healing [48] with some modifications (Fig. 1A). Briefly, mice were anesthetized with tribromoethyl alcohol (20 mg/mL, 10 μL/g injection), and the hair was shaved and cleaned with 70% ethanol. We split the back skin with a ring of medical silica gel membrane (to prevent the wound closure due to loose skin of mice), and then seamed it well with medical thread and removed the center skin to create 10 mm full-thickness excisional dorsal skin wounds using a sterile scissor. Wound areas were calculated using ImageJ software, and the wound closure percentage was obtained as follows:

$$\textrm{Wound}\ \textrm{closure}\ \textrm{percentage}\ \left(\%\right)=\frac{\textrm{wound}\ \textrm{area}\ \textrm{on}\ \textrm{day}\ 0-\textrm{wound}\ \textrm{area}\ \textrm{on}\ \textrm{day}\ \textrm{n}}{\textrm{wound}\ \textrm{area}\ \textrm{on}\ \textrm{day}\ 0}\times 100\%$$

### Muscle contusion model

We developed a muscle contusion model as stated in the reference [49] to explore the role of hydrogen in muscle injury repair. Male C57/BJ mice aged 6–8 weeks were used. The detailed method is to drop a 25-g weight from a height of 60 cm to the inner surface of the gastrocnemius muscle (Figure S9A). This modeling method has moderate intensity and will not cause skeletal injury or gait abnormality.

### H_2_ chamber treatment

A transparent closed box (20 cm × 18 cm × 15 cm) was connected to a hydrogen generator (KLE-H7, Kelieng Biomedical Co. Ltd., Shenzhen, China) (Fig. 1A), which produces 66% H_2_ and 33% O_2_ (V/V), 5% H_2_ (V/V) mixed with air, or 33% O_2_ (V/V) mixed with air. Hydrogen treatment was given immediately after establishment of the mouse cutaneous wound healing establishment, and then daily administration was given until the end of the experiment. Animals were placed in the box with the mixed air for 1 h each day. During this inhalation period, mice were awake and freely moving. Thermal trace GC ultra-gas chromatography (Thermo Fisher, MA, USA) was used to monitor the concentration of hydrogen gas in the closed box.

### H_2_ rich medium preparation

H_2_ rich medium was produced by injecting gas using the same hydrogen generator used for H_2_ Chamber treatment (KLE-H7, Kelieng Biomedical Co. Ltd., Shenzhen, China) into DMEM medium (Gibco) for 30 min hydrogen dissolved in reaction solution was detected with a needle-type H_2_ sensor (Unisense, Aarhus N, Denmark). To measure the H_2_ concentration in the reaction system, the sensor was inserted below the liquid surface.

### Blood perfusion and blood oxygen detection

Blood perfusion at the wound site during the healing processes was detected by using a moorFLPI-2 (Moor Instruments Limited, UK) according to manufacturer instructions. Images were further selected from videos and analyzed by moorFLPIReview V50 software (Moor Instruments Limited, UK). Blood oxygen levels were tested by the moorVMS-OXY Tissue Oxygen Monitor (Moor Instruments Limited, UK) according to the instructions. Five sites (top, bottom, left, right, and middle) of the wound area were chosen, and an average was calculated to create the oxygen kinetic curve.

### In vitro tube formation assay

Tube formation assay was performed as described [50]. Firstly, Matrigel was thawed at 4 °C overnight to avoid bubble formation. Briefly, 15-well μ-Slides (ibidi, Germany) were coated with 10 μl of Matrigel, which was allowed to solidify at 37 °C. HUVECs before generation 10 were harvested and the cell number and viability were determined by Trypan blue staining before seeding. A cell suspension was prepared in DMEM and 15,000 cells/well were seeded in 50 μl medium on top of the matrigel. HUVECs were seeded in conditioned medium for 6 h, and the enclosed networks of complete tubes were counted and photographed under an inverted microscope. The tubular loops of the cells were measured and calculated for each well.

### Digital Spatial Profiler and bulk RNA-seq analysis

Bulk RNA-seq was processed by Anoroad Inc. (Beijing, China) and the Digital Spatial Profiler assay was performed by Capital Bio Technology Inc. (Beijing, China).

### Immunohistochemistry (IHC) and immunofluorescence (IF)

#### Tissue immunostaining

Skin tissue at the wound site was harvested and fixed with formalin and embedded in paraffin blocks, and then 4 μm thick paraffin sections were mounted on glass slides for histological staining. IHC and IF of the paraffin-embedded tissue sections were performed as previously indicated [51]. Briefly, sections were dewaxed and rehydrated by subsequent immersion in xylene, ethanol (100%, 95%, 70%, and 50%) and deionized H_2_O. Antigen was then retrieved in citrate buffer, and non-specific staining between the primary antibodies (Table S1A) and the tissue was blocked by incubation in 1% goat serum in PBS for 60 min at RT. The sections were incubated with the primary antibodies listed in the Table S8A at 37 °C for 60 min for IHC or at 4 °C overnight for IF. Further labeling with specific secondary antibodies for IHC or IF (Table S1B) was performed according to the manufacturer’s instructions. For IF, nuclei were stained with NucBlue Live cell stain (R37605, Invitrogen). DAB, hematoxylin and neutral balsam mounting reagent used in IHC processes were obtained from ZSGB-BIO (Beijing, China).

#### Cells immunofluorescence staining

Cells suspensions in a 96-well plates at 10,000 cells/well were allowed to adhere for 24 h and then switched to full medium. Wells containing the seeded cells were washed with 1× PBS immediately at the end of the incubation times and fixed in 4% paraformaldehyde for 30 min. Subsequent to the fixation, the cells were permeabilized with 0.3% PBS-Tween for 15 min, washed, and then blocked with normal sheep serum blocking buffer (ZSGB-BIO, China) for at least 1 h at room temperature. Primary antibodies (Table S1A) were added at the dilution recommended by the manufacturers and were incubated overnight at 4 °C. Appropriate fluorescent dye-labeled secondary antibodies (Table S1B) were used, and cell nuclei were stained with NucBlue Live cell stain (R37605, Invitrogen).

### Hematoxylin and eosin and Masson trichrome staining

The protocol described by Fischer et al was used for the Hematoxylin and eosin (H&E) staining [52], while Masson trichrome staining was performed in strict accordance with the manufacturer’s protocol (Masson Stain Kit (60532ES58), Yeasen, China).

### Flow cytometric staining

For analysis of Th_1_, Th_2_, *T*_reg_, and Th_17_ cells, single-cell suspensions were prepared from the spleens of mice 24 h after wounding. The cells were labeled with CD8-APC, CD4-FITC, and CD3-PECy5. For T-bet and GATA-3, protein amounts were normalized, and cell surface staining was performed using APC-conjugated anti-CD4 followed by fixation with 1× Fixation/Permeabilization buffer and intracellular staining with PE-conjugated anti–T-bet and APC–conjugated anti–GATA-3 in 1× permeabilization buffer. Cells were washed in 1× permeabilization buffer and analyzed by flow cytometry (B53009, CytoFLEX Flow Cytometer, Biotek).

For the analysis of *T*_reg_ cells, cell surface staining was performed using FITC-conjugated anti-CD4, PE-conjugated anti-CD25, PB450-conjugated anti-CD127 and appropriate isotype controls. Cells were incubated with antibodies for 20 min at room temperature in the dark, followed by washing in phosphate buffered solution (PBS) and analysis by flow cytometry. For the analysis of Th_17_ cells, cells were stained with FITC anti-CD4 for surface expression of CD4, and intracellular cytokine IL-17 was detected by staining with APC-conjugated Anti-IL-17. Finally, cells were analyzed by flow cytometry. All antibodies and reagents were purchased from Biolegend Inc., USA (Table S1).

For the identification of MSCs, 8 conjugated antibodies (CD73-PE, CD90-FITC, CD105-Cy5, CD34-FITC, CD45-Cy5, CD79a-PE, CD14-APC, and HLA-DR-APC-Cy7) targeting at the cell surface markers [53] were applied, and the staining and detection methods were consistent with the methods described previously.

### Enzyme-linked immunosorbent assay

Murine serum was carefully collected from whole blood and stored in −80°C. Wound edge tissue was harvested after the mice were sacrificed, washed twice in distilled PBS, and cut into small pieces in lysis buffer containing protease inhibitor and 0.1 mM PMSF (Sangon Biotech, Shanghai, China), followed by homogenization and centrifugation (12,000 rpm, 20 min). The supernatant was then collected for further assessment of EGF (MM-0043M), bFGF (MM-0050M1), PDGF (MM-0070M1), and TGF-β1 (MM-0921M) levels using mouse ELISA kits (Jiangsu Meimian industrial, China). Tissue and serum samples underwent multiple cytokines detection by using Bio-plex pro^TM^ Mouse Cytokine Th17 Panel A 6-Plex kit (#M6000007NY, Bio Rad, USA). For tissue detection, amounts of target growth factors were normalized to the total amount of whole protein.

### Non-targeted metabolomics analysis

The non-targeted metabolomics analysis was performed by IGENECODE Company, Beijing, China. Thermo Scientific™ Dionex™ UltiMate™ 3000 Rapid Separation LC (RSLC) system. UHPLC separation was achieved with reverse phase C18 or hydrophilic interaction liquid chromatography columns. For C18 separation, the mobile phase A was acetonitrile/water (60/40) and mobile phase B was isopropanol/ acetonitrile (90/10); both A and B contained 0.1% formic acid and 10 mmol/L ammonium acetate. The gradient conditions for reverse phase C18 separation are shown in Table S2. The HSS T3 column (2.1 × 100 mm, 1.8 μm, waters) operated at 45 °C. The flow rate was 300 μL/min, and the injection volume was 1 μL.

For HILIC separation, the mobile phase A was acetonitrile, and the mobile phase B was water; both A and B contained 0.1% formic acid and 10 mmol/L ammonium acetate. A BEH Amide column (2.1 × 100 mm, 1.7 μm, waters) was operated at 40 °C. The flow rate was 300 μL/min, and the injection volume was 1 μL (see Table S3).

A Thermo Scientific™ Q Exactive™ hybrid quadrupole Orbitrap mass spectrometer equipped with a HESI-II probe was employed. The pos HESI-II spray voltage was 3.7 kV, the heated capillary temperature was 320 °C, the sheath gas pressure was 30 psi, the auxiliary gas setting was 10 psi, and the heated vaporizer temperature was 300 °C. Both the sheath gas and auxiliary gas were nitrogen. The collision gas was also nitrogen at a pressure of 1.5 mTorr. The parameters of the full mass scans were as follows: a resolution of 70,000, an auto gain control target under 1 × 10^6^, a maximum isolation time of 50 ms, and an *m/z* range 50–1500. The LC-MS system was controlled using Xcalibur 2.2 SP1.48 software (Thermo Fisher Scientific), and data were collected and processed with the same software.

All data obtained from the four assays in the two systems in both pos and neg ion modes were processed using Progenesis QI data analysis software (Nonlinear Dynamics, Newcastle, UK) for imputing raw data, peak alignment, picking, and normalization to produce peak intensities for retention time (tR) and m/z data pairs. The ranges of automatic peak picking for the C18 were between 1 and 16 min and between 1 and 12 min, respectively; adduct ions of each “feature” (m/z, tR) were deconvoluted, and these features were identified in the human metabolome database (HMDB) and Lipidmaps.

### Fibroblast movement and migration and keratinocyte cell epithelialization ability test by a live cell imaging system

HPF cells were seeded at 5000 cells/well in a 96-well plate with a clear bottom, stained with Actin-GFP (C10506, CellLingt, Invitrogen, USA) and cultured for another 24 h to observe movement. ESF cells were seeded at 20,000 cells/well, scratched by the high throughput scratcher of Cytation 5 (Biotek), and then observed for another 24 h to determine the conditioned medium in fibroblast migration function. Cell movement and migration were scanned using a Cytation 5 Cell Imaging Multi-mode reader (Biotek), and migration was quantified automatically according to the manufacturer’s instructions. HaCaT cells were seeded at 30,000 cells/well and then cultured for another 24 h to observed and calculate the area of colony formation (epithelialization). The images in each small field were captured every hour in 3 × 3 montage frames at × 10 magnification. For cell movement video footage, both bright field and fluorescent channel were used.

### Preparation and evaluation of magnesium based hydrogen storage material dressing

Microparticles of magnesium (Mg) balls (average diameter 20 μm) are spread evenly on medical gauze soaked with physiological saline solution. Medical waterproof and breathable polyurethane film was covered on both sides of the medical gauze. Then the “sandwich dressing” was sealed around the edge, and sewed onto the silicone membrane at the back of the wounded animal. For the control group, normal medical gauze soaked with normal saline solution was used without Mg. All the dressings were changed every day. The release of H_2_ was measured by headspace gas chromatography (GC) (Shimadzu, GCMS-QP2010S).

### Statistical analysis

For comparison between two groups, two-tailed paired and unpaired Student’s *t* tests were performed to calculate *P* values and determine statistically significant differences (significance was set at *P* < 0.05, as detailed in the figure legends). For comparison among more than two groups, ordinary one- or two-way analysis of variance (ANOVA) tests were followed by the appropriate multiple comparison tests (as detailed in the figure legends). All experiments were repeated twice with the same results. All statistical analyses were performed with GraphPad Prism 8 software.

## Results

### Hydrogen greatly accelerates cutaneous aseptic wound closure in full-thickness dorsal-skin defect mice

To determine whether H_2_ improves wound healing, we established a cutaneous aseptic wound model using full-thickness dorsal-skin defect mice that were subjected to daily application of H_2_ in a treatment chamber (Fig. 1A). Sampling region and the scheme of wound were also shown in Fig. 1A. We evaluated the effect of high (66% H_2_, 66% H_2_ + 33% O_2_) and low (5% H_2_; 5% H_2_ + 21% O_2_ air) concentrations of H_2_ on wound healing over an 11-day time course (Fig. 1B), and included control (air) and 33% O_2_ (33% O_2_ in air) mouse groups for comparison_._ H_2_ has long been recognized as an antioxidant targeting ROS; therefore, we also added an N-acetyl-L-cysteine (NAC) treatment group to evaluate a possible role of ROS cleavage in wound healing. Our results revealed that the 66% H_2_ group (66% H_2_ + 33% O_2_) healed faster than any of the other groups. Macroscopic differences appeared on the first day after treatment and became more visible on day 3 (Fig. 1C). Eleven days after wounding, the 66% H_2_ group displayed a significantly increased wound-closure rate (approximately 90% wound closure for the 66% H_2_ group; as compared to 70% closure for the 5% H_2_ group and about 30% closure for the control group; Fig. 1D). Furthermore, the healing rates of the NAC and 33% O_2_ groups were similar to those of the control group. These results suggest that H_2_ promotes healing in a dose-dependent manner, and that the healing effect is not dependent on O_2_ or ROS (Fig. 1C, D).

To further visualize the effect of H_2_ on wound healing, we performed histological analysis of haematoxylin and eosin (H&E) and Masson-stained sections on day 11 (Fig. 1E). The localization of the wounded skin tissue is shown in the schematic diagram (Fig. 1A, right part). The epidermis was more fully formed, and the dermal and epidermal junction (DEJ) appeared intact with more collagen deposition in the 66% H_2_ group, while a weaker DEJ and partial re-epithelialization were observed in both the NAC and 33% O_2_ groups. This is despite the effect of NAC in reducing EGF and TGF-β1 growth factor levels at day 3 after wounding (p_bFGF_ < 0.01, p_TGF-β1_ < 0.05) (Figure S1). Thus, these results verify that neither ROS cleavage nor 33% O_2_ administration alone were able to promote the wound healing process. Notably, the wound healing pattern in the 66% H_2_ group appears similar to moist healing, with less blood scab formation and better in tissue remodeling.

As the benefits of 66% H_2_ application on wound healing were visible on day 3, we evaluated whether abbreviated treatment might have therapeutic value. For these experiments, we treated mice with 66% H_2_ for 3 days only, and then stopped administration while continuing to monitor the mice for another 6 days. As shown in Fig. 1F, G, the 66% H_2_ group healed faster than the control group; however, all healing speeds were much slower, with final wound closer rates of < 50% and blood clots visible after days 3–5. Therefore, these results suggest that persistent daily treatment is required for the full benefits of H_2_.

### Hydrogen increases blood flow and blood oxygen levels in the wound area and promotes early blood vessel formation

**Fig. 2:**
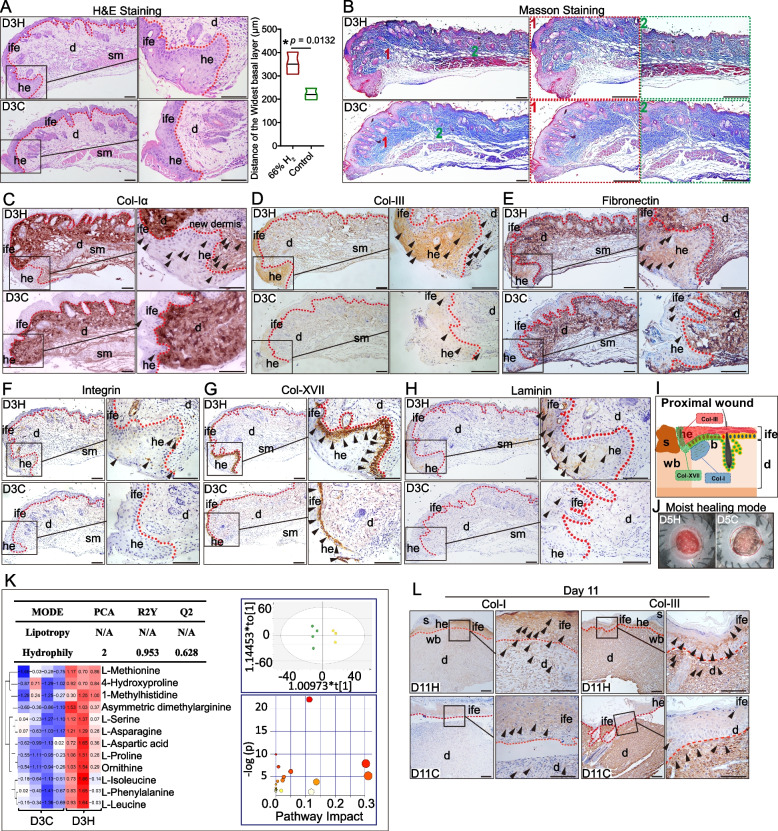
H_2_ treatment promoted different ECM components early deposit in the epidermis, dermis and dermis-epidermis-junction of proximal wound. **A** Representative H&E staining of the truncated region of epidermis at the wound edge at 3 days post-wounding between two groups; graph indicated the widest basal layer of hypertrophic epidermal wound edge. **B** Masson staining of different regions of wound edge at 3 days post-wounding between two groups. **C**–**H** IHC staining showing the expression of ECM components of Col-I, Col-III, Fibronectin, Integrin, Col-XVII, and Laminin, respectively in the wound edge between D3H and D3C groups. **I** Scheme of a proximal wound section indicating the histological localization of three kinds of collagens: Col-I (dermis), Col-III (epidermis), and Col-XVII (DEJ) expression after H_2_ treatment during the re-epithelialization process. **J** Representative image of day 5 post-wounding indicating the moist haling mode possibly triggered by the early ECM deposition induced by H_2_ treatment. **K** Overview of mapping of metabolic differences between the 66% H_2_ and control groups as well as PCA analysis and pathway enrichment of differential metabolomes; *R*^*2*^_*X*_ = fraction of variance for the model; *Q*^*2*^ = predictive ability of the model. **L** IHC staining showing the expression of Col-I and Col-III between D11H and D11C groups. Data in **A** processed unpaired *t* test, and were plotted as Mean ± SEM. *P value < 0.05; **P value < 0.01; ***P value < 0.001; no stars for *P* value > 0.05; scale bar = 100 μm. Red line indicates the boundary between the epithelium and dermis. Black arrowhead indicates positive-expressing cell. d, dermis; he, hypertrophic epidermal wound edge; ife, interfollicular epithelium; sm, smooth muscle

As important indicators of wound healing, the wound blood flow and blood oxygen [54, 55] content are tested to evaluated the effects of 66% H_2_ on perfusion. From day 5 after wounding until the final day of imaging, blood perfusion in the wound bed was significantly higher in the 66% H_2_ group that in the other groups (*P* [66% H_2_ vs. control] < 0.0001) (Fig. 1H, I), indicating better tissue survival and tube formation [56]. Furthermore, the oxygen saturation (SaO_2_), total haemoglobin mass (tHb), and deoxyhaemoglobin (HHb) values were all higher starting at day 3 in the 66% H_2_ group than in the 5% H_2_ and control groups (Fig. 2I). The initiation of vessel formation by H_2_ may happen earlier than day 5 post-wounding.

CD31 staining 11 days after wounding suggested the 66% H_2_ group had the most pronounced tube formation (more than six times compared with that in the control group) (Fig. 1J, K). Whole-mounting scans targeting CD31 suggested that, from day 3 onward, 66% H_2_ already induces visible vessel formation, especially in the proximal part of the wound site under the epidermis (Fig. 1L, M, Figure S2). In vitro tube formation test showed that the 66% H_2_ group had a similar effect as the positive control group (bFGF) (Fig. 1N, O), and better than the 5% H_2_ (with air), N_2_ (no air), and 33% O_2_ (No H_2_) (Figure S3 A, B). Tube formation-related genes were upregulated in D1H but downregulated in D3H (Fig. 2P), indicating an early activation of tube formation by 66% H_2_. Further whole-mount tissue scanning suggested H_2_ accelerated the visible vessel formation of proximal wound since day 2 post-wounding (Figure S3A, B). These results are consistent with a role for H_2_ in early vascularization, which could contribute to wound healing.

### Hydrogen induced transcriptomic signatures associated with early ECM deposit, tube formation, cell migration and differentiation, and mitochondrial repair

Given the remarkable healing by 66% H_2_ at day 3 after wounding (the epithelialization phase), we performed functional annotation and enrichment analysis of RNA-seq data from skin samples to identify underlying processes. Based on differences in expression between the 66% H_2_ and control group (see the total DEGs in Table S4), 68 differentially expressed genes (DEGs) from D1H (day 1 H_2_) vs D1C (day 1 control) (58/10, up/down) (Table S5), 18 DEGs from D2H vs D2C (12/6, up/down) (Table S6), and 24 DEGs from D3H vs D3C (18/6, up/down) (Table S7) were identified (adjusted *p* value ≤ 0.01 and │FC│ ≥ 1.5), with no overlap between the sets of DEGs (Figure S4A–C). Heatmap cluster analysis of all DEGs identified 4 clusters (Figure S4D). Among the top-10 Gene Ontology Biological Processes (Figure S4E) in cluster I, the DEGs were mainly enriched in hormone response and signal transduction processes, which were upregulated mainly in D2H; genes in cluster II were enriched mostly in biological functions related to tube formation, cell migration and extracellular matrix (ECM) organization and were upregulated first in D1H, then gradually decreased to an equivalent expression level between D2H and D2C, and upregulated again in D3C; genes enriched in cluster III were mostly related to muscle cell differentiation and organization and were upregulated in the D3H group; and genes in cluster IV were involved in processes related to metabolism and were upregulated in D2C. Gene set enrichment analysis (GSEA) confirmed the pattern of DEG enrichment in ECM deposition-, cell adhesion-, and tube formation-related function in D1H (Figure S4F); muscle filament sliding and myofibril assembly related functions in D2H (Figure S4G); and myocyte migration and differentiation and mitochondria activity-related functions in D3H (Figure S4H).

### Hydrogen induces early ECM deposition during the epithelialization process

Considering of the important role of ECM in epithelialization [12], the effect of 66% H_2_ on early ECM deposition at 1–3 days after wounding was evaluated, which is when the proliferative phase normally starts and keratinocytes migrate into the wound bed. H&E staining showed a thicker epidermal layer on the hypertrophic epidermal wound edge in the D3H group as compared to the D3C group (*p* = 0.0132) (Fig. 2A). Furthermore, Masson staining revealed more dermal collagen deposition in the D3H group at both proximal and distal wounds (Fig. 2B). Consistently, immunohistochemical staining (IHC) showed that col-I (Fig. 2C) and col-III (Fig. 2D) were more highly expressed in the D3H group, especially around the proximal area of the wound. Fibronectin (Fig. 2E), integrin (Fig. 2F), col-XVII (Fig. 2G), and laminin (Fig. 2H) were also elevated in the D3H proximal epidermis, indicating better DEJ formation after H_2_ treatment. Scheme of the col-I, col-III, and col-XVII expression in the proximal wound edge was shown in Fig. 2I. ECM deposition may contribute to maintain the moist healing (Fig. 2J).

As additional evidence for the importance of the ECM, we examined the differential expression of metabolites. Twelve metabolites (Table S8, S9), all of which were amino acids, were differentially expressed on day 3 (Fig. 2K). Among them, L-proline, and especially 4-hydroxyproline, are known to provide raw materials for collagen synthesis [57]. Pathway categorization supported an increase in metabolites thought to contribute to an environment that supports ECM deposition in the wound bed after H_2_ treatment (Table S10). Furthermore, correlation analysis of D3 non-target metabolism and RNA-seq showed similarly enriched genes in tight junctions and focal adhesion pathways (Figure S5).

We also examined ECM deposition at the wound site on day 2 by IHC, which demonstrated that col-I, fibronectin and laminin were more highly expressed in the D2H group than in the D2C group, at the proximal wound (Figure S6A–C), though no difference was observed in integrin expression (Figure S6D). This deposition of collagens in the 66% H_2_ group lead all the way to day 11 post-wounding, especially for dermal Col-1 and epidermal Col-III (Fig. 2L).

### Hydrogen induces α-SMA^+^/K14^+^ keratinocytes and α-SMA^+^ myofibroblast migration to the wound edge

**Fig. 3:**
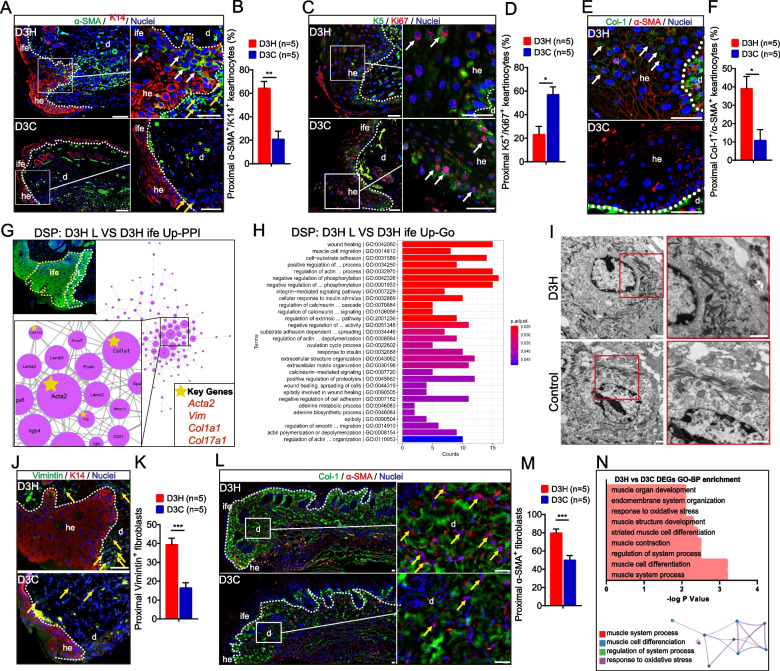
H_2_ induces myofibroblasts and fibroblasts migration and IFE towards α-SMA^+^ myoepithelial cell transformation in the proximal wound. **A**, **C**, **E**, **J** Detection of α-SMA/K14, K5/ki67, Col-1/α-SMA and Vimintin/K14 in the leading edge of the proximal wound at day 3 post-wounding. **L** Col-1/α-SMA co-expression in the dermal fibroblast of proximal wound at day 3 post-wounding. **B**, **D**, **F**, **K** and **M** quantification of double/single positive cells in **A**, **C**, **E**, **J** and **M**. **G**, **H** DSP transcriptome analysis of D3H proximal wound area (D3H leading edge vs D3H ife) showing the upregulated PPI network, key genes and up-GO biological processes in the keratinocytes of leading edge. White and yellow dotted circles indicate ROI of leading edge and ife, respectively. **I** Observation of keratinocyte tonofilament morphology in 66% H_2_ and control groups by electron microscope. **N** GO-BP enrichment and enriched GO-BP clusters of D3H vs D3C DEGs by using metascape online analysis. White dotted line indicates the boundary between the epithelium and dermis. White arrows in **A**, **C,** and **E** indicate co-positive IFE cells, yellow arrows indicate Vimintin^+^ (**J**) fibroblasts or Col-1^+^/α-SMA^+^ co-positive myofibroblasts (**L**). Data in **B, D, F, K**, and **M** processed Unpaired T test, and were plotted as Mean ± SEM. *P value < 0.05; **P value < 0.01; ***P value < 0.001; no stars for *P* value > 0.05; Scale bar in **A**, **C**, **E**, **J**, and **L** = 100 μm; Scale bar in **I** = 1 μm. d, dermis; he, hypertrophic epidermal wound edge; ife, interfollicular epithelium. DSP, digital spatial profiler

**Fig. 4:**
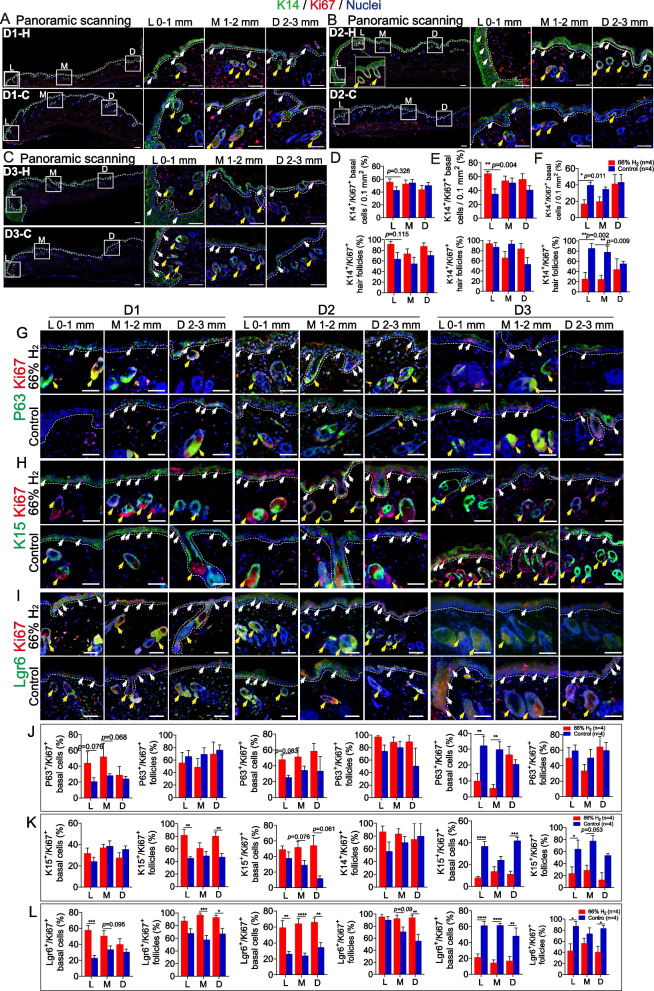
H_2_ treatment promoted in vivo early epidermal stem cells proliferation at days 1–3 after wounding. **A**-**C** Panoramic scanning of wound edge and the representative immunofluorescence images of K14 (green) and ki67 (red) expression in the leading edge (L, 0–1 mm from wound edge), mid-end (M, 1−2 mm from wound edge) and distal end (D, 2-3 mm from wound edge) of the wound; **D**−**F** Statistics of K14^+^/ki67^+^ basal cells and hair follicles in the immunofluorescence. **G-I**. Representative immunofluorescence of p63 (green) and ki67 (red), K15 (green) and ki67 (red), and Lgr6 (green) and ki67 (red) on skin section showing their expression in the leading edge (L), mid-end (M) and distal (D) of the proximal wound in epidermal and hair follicle cells from 1 to 3 days after wound. **J**−**L**. Statistics of p63^+^/ki67^+^, K15^+^/ki67^+^, and Lgr6^+^/ki67^+^ basal cells and hair follicles in the immunofluorescence from 1 to 3 days after wounding. White arrow indicates co-positive basal cell; yellow arrow indicates co-positive follicle cell. Data in **D–F**, **J–L** processed two-way ANOVA test, and were plotted as Mean ± SEM. * *P* value < 0.05; **P value < 0.01; ***P value < 0.001; no stars for *P* value > 0.05; Scale bar = 100 μm

Collagen and other ECM components can induce keratinocyte migration [58, 59]. To evaluate the effect of H_2_ on epithelialization at the proximal wound edge (leading edge), we performed immunofluorescent staining of markers for myoepithelial cells/myofibroblasts (α-SMA), keratinocytes (K14), and IFE cells (K5). Day 3 whole-mount staining demonstrated more than 3 times the amount of α-SMA^+^/K14^+^ keratinocytes in the D3H group as compared to the control group (especially in the migration tongue region of hypertrophic epidermal leading edge) (Fig. 3A, B), indicating transformation from keratinocytes to a myoepithelial-like cell type. However, the IFE keratinocytes displayed slower proliferation rates in the D3H group than in the D3C group (Fig. 3C, D, *p* < 0.05), which may be due to a more advanced cell proliferation induced by H_2_. Although not highly expressed, the epidermal keratinocytes at the leading edge produced more col-1 (α-SMA^+^/Col-1^+^) in the D3H group than in the D3C group (Fig. 3E, F). The transformation direction from keratinocytes to myoepithelial cells, were then proved by digital spatial profiler transcriptome analysis (D3H Leading edge vs D3H ife of the proximal wound). Seeing from Fig. 3G, key genes selected after up-PPI analysis include Acta2 (α-SMA), Vim (Vimintin), Col1a1 (col-I), and Col17a1 (col-XVII). D3H L up-GO BP were mostly enriched in myoepithelial cell-related functions (Fig. 3H). Electron microscopy demonstrated a higher density of tonofilaments arranged around the plasma membrane of keratinocytes at the leading edge of the D3H group (Fig. 4I); the correlation analysis of transcriptomics and metabolomics also revealed that, the focal adhesion-related function was enriched in D3H group (Figure S5). These results could support better migration ability for myoepithelial-transformed keratinocytes [60]. Furthermore, both and Vimentin^+^ fibroblasts (Fig. 3J, K, *p* < 0.001) and α-SMA^+^ myofibroblasts (Fig. 3L, M, *p* < 0.001), which are two of the main sources of collagen, were more abundant in the proximal wound of the D3H group. In vitro IF staining also proved that fibroblasts (HPF, (Figure S7E)) and keratinocytes (HaCat, Figure S7B) were producing more col-1 after H_2_ treatment. Besides, the primary cell HUCP-MSCs have been identified to maintain the MSCs phenotypes (Figure S7A, B). H_2_ promoted the HUCP-MSCs transformation into a myofibroblast phenotype (Vemintin+, α-SMA+) (Supplemental Figure S7C) with better collagens producing (Supplemental Figure S7C) abilities. Consistent with all the staining results, transcriptome analysis of D3 identified top Gene Ontology biological process (GO-BP) enrichment categories involved muscle cell development, differentiation, and contraction (Fig. 3N).

### Hydrogen induces early IFE and HF stem cells proliferation

IFE stem cells (IFESCs) are thought to promote wound healing and replace irreversibly lost skin [61] , while hair follicle stem cells (HFSCs) around the wound replenish the basal layer and reconstitute non-proliferative, transcriptionally active spinous and granular layers [17, 62]. To characterize the early proliferation and distribution of IFESCs and HFSCs in early wounding, we performed whole mount tissue scans. More k14^+^/Ki67^+^ basal IFESCs were identified in the D1H (Fig. 4A, D) and D2H groups (Fig. 4B, E), particularly at the leading edge (L, 0–1 mm from the wound site). Furthermore, the proportion of k14^+^/Ki67^+^ HFs was more abundant in the D1H group in the L, M (middle), and D (distal) edges of the wound site (Fig. 4D). However, 3 days after wounding, there were fewer k14^+^/Ki67^+^ basal cells and HFs in the D3H group, especially in the L and M parts of the wound (Fig. 4C, F), indicating an advanced activation for the IFESCs proliferation triggered by H_2_.

We further characterized IFESCs and HFSCs by examining the expression of specific markers in proliferating cells after H_2_ treatment. The results demonstrate that p63^+^/Ki67^+^ basal IFE cells were more abundant in the D1H and D2H groups in the L, M, and D parts of the proximal wound after H_2_ treatment (Fig. 4G, J). Additionally, there were more k15^+^/Ki67^+^ hair follicles in the D1H group and k15^+^/Ki67^+^ basal IFE cells in the M and D wound in the D2H group (Fig. 4H, K). There were also more Lgr6^+^/Ki67^+^ basal IFE cells in the D1H and D2H groups in the L, M, and D parts of the wound site (Fig. 4I, L), while Lgr6^+^/Ki67^+^ HFs were more abundant in the M and D parts in the D1H and D2H groups (Fig. 4I, L). All p63^+^/Ki67^+^, k15^+^/Ki67^+^, and Lgr6^+^/Ki67^+^ basal IFE cells and HFs were decreased for the first 3 days after H_2_ treatment (Fig. 6J–L). Thus, H_2_ treatment induces early proliferation of basal IFE stem cells and HFSCs that contribute to epidermal thickening and wound closure.

### Hydrogen alleviates inflammatory responses by reducing especially the Th_1_ and Th_17_ population during aseptic wound healing

Inflammation also influences wound healing and matrix deposition [63, 64]. Therefore, we sought to determine the effect of 66% H_2_ treatment on Th cell subsets. Flow cytometry of mouse spleens 24 h after wounding showed the CD4^+^ and CD8^+^ T cells had no significant differences among the three groups (Figure S8A), while the population of Th_1_ and Th_17_ subgroups were reduced by H_2_ treatment (Figure S8B, D). No significant differences were observed in the other two subsets of Th_2_ and Treg cells (Figure S8C, E). A representative gating strategy was shown in Figure S8F. The splenic FCM detection revealed H_2_ alleviated Th_1_- and Th_17_-related systemic inflammation during the early stage of wound healing. Due to the aseptic wound (daily oral intake enrofloxacin for each group) [65], tissue cytokine profile for the H_2_ group has no big difference with the control and NAC group, though TNF-α, IL-1β were reduced and IL-10 was slightly increased 24 h after wounding in the 66% H_2_ group (Figure S8G).

### H_2_ promoted keratinocytes in re-epithelialization and fibroblasts/MSCs in migration possibly due to maintaining the mitochondrial functions

**Fig. 5:**
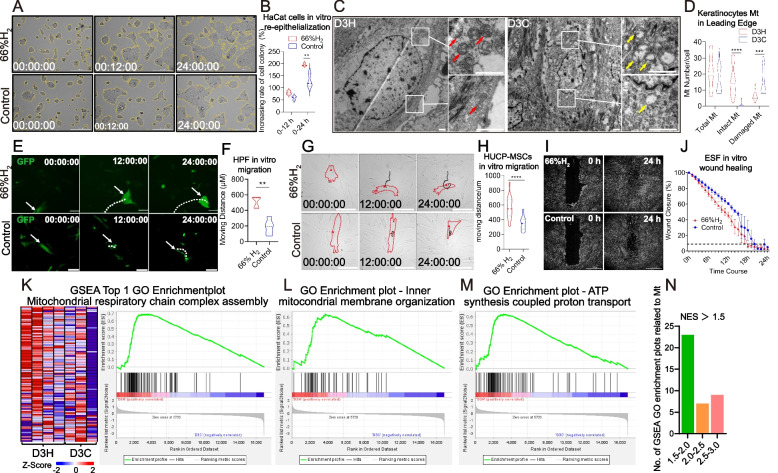
H_2_ promoted *in vivo* and *in vitro* keratinocytes and fibroblasts cells viability by accelerating the re-epithelialization, migration and mitochondrial functions. **A**. Representative images of HaCat cell epithelization. Images were captured every two hours using a live cell imaging system, and the 0, 12 and 24 hour images were presented. **B**. The increased rate of the total colony area in 12 and 24 hours. **C**. Comparison of mitochondrial morphology 3 days post-wound for the D3H and D3C groups observed by electron microscope. The right two images in each panel are enlargements of the white frames in the left images. Red arrows indicate intact mitochondria, while yellow arrows indicate damaged mitochondria. **D**. Comparison of total mitochondria, intact mitochondria and damaged mitochondria numbers between D3H and D3C groups. **E** and **G**. Representative images of HUCP-MSCs and HPF fibroblasts migration at 0-h, 12-h, and 24-h time points in H_2_ medium and control groups. **F**, **H** Graphic analysis of HUCP-MSCs and HPF fibroblasts total moving distance in 24 h. **I**. Cell migration ability examined by wound-healing assay in H_2_ medium and control groups. **J** Wound-healing curve analysis under different conditions. **K** Heatmap and enrichment plot showing genes related to mitochondrial respiratory chain complex assembly, which is the top 1 GO enrichment function in D3H. **L**, **M** Top GO enrichment plots in D3H related to inner mitochondrial membrane organization and ATP synthesis-coupled proton transport, which indicates more intact and functional mitochondria in D3H. **N** GSEA GO enrichment plots enriched in Mt related functions for D3H (NES > 1.5). White and black dotted lines in **E** and **G** indicate the migration route of fibroblasts. Red dotted line in **G**. outlines the representative HUCP-MSCs. Data in **B** and **D** processed two-way ANOVA test, and data in **F**, **H**, and **J** processed unpaired *t* test. Data were plotted as mean ± SEM. *P value < 0.05; **P value <0.01; ***P value < 0.001; no stars for *P* value > 0.05; Scale bar in A and G = 200 μm, scale bar in C = 2 μm, scale bar in E = 100 μm, scale bar in I = 1000 μm

In the re-epithelialization phase of wound healing, epithelial cells migrate to the wound site, cover the granulation tissue, and then converge in the middle. In vitro live cell imaging revealed an enlarged HaCat cell colony area in the H_2_ group (Fig. 5A, B). Furthermore, we performed electron microscopy 3 days after wounding and observed the Mt of the keratinocytes in the leading edge (0–1 mm from wound edge). The D3H group showed increased total Mt numbers, with more intact and fewer damaged Mt (Fig. 5C, D).

To confirm the cell migration ability enhanced by H_2_, HPF fibroblasts and HUCP-MSCs were plated and observed by live cell imaging system for 24 h after H_2_ medium treatment. For the HPF cells, the total moving distance in the H_2_ group was 2.5 times higher (*p* < 0.01) than that in the control group (Fig. 5E, F; videos provided in supplemental movies 1 and 2). Similarly, at 24 h, the total moving distance of HUCP-MSCs were significantly higher in the H_2_ group than in the control group (*p* < 0.0001) (Fig. 5G, H). In a live cell scratch assay, human embryonic skin fibroblast ESF cells healed faster in the H_2_ group than in the control group (Fig. 5I, J).

The GSEA from D3H vs D3C up-Go genes were enriched in mitochondria (Mt) structure and function (Figure S4H), which are important for cell migration. Detailed GO-plots related to Mt organization and energy generation were extracted for the D3H up-GO GSEA (Fig. 5K–N). All these mitochondrial functions promoted by H_2_ give promise to a better cell viability for keratinocytes, fibroblasts, and MSCs in the wound edge.

### H_2_ promote the early repair of muscle and skin nerve injuries

Since hydrogen can promote early epithelization and tissue remodelling during wound healing processes, it is also likely to have a good repair effect on other tissue injuries such as muscle and nerve. In the muscle contusion model (Figure S9A), the swelling and blood perfusion of gastrocnemius muscle was significantly relieved in the 66% H_2_ group since day 2 onward (Figure S9B-D). These results indicated early repair functions of H_2_ in soft tissue muscle damage. On the other hand, NFH (neurofilament, heavy polypeptide) was used as a marker to reveal the possible function of hydrogen in nerve injury repair and axons maturation. More NFH^+^ cells were observed in the 66% H_2_ group of proximal wound edge on day 3 and day 12 post-wounding, suggesting the cutaneous nerve was repaired by H_2_ treatment at an early stage. Other kinds and more complicated tissue injury restoration will be further explored in our future research.

### Topical treatment of H_2_-releasing dressing promoted wound healing

**Fig. 6:**
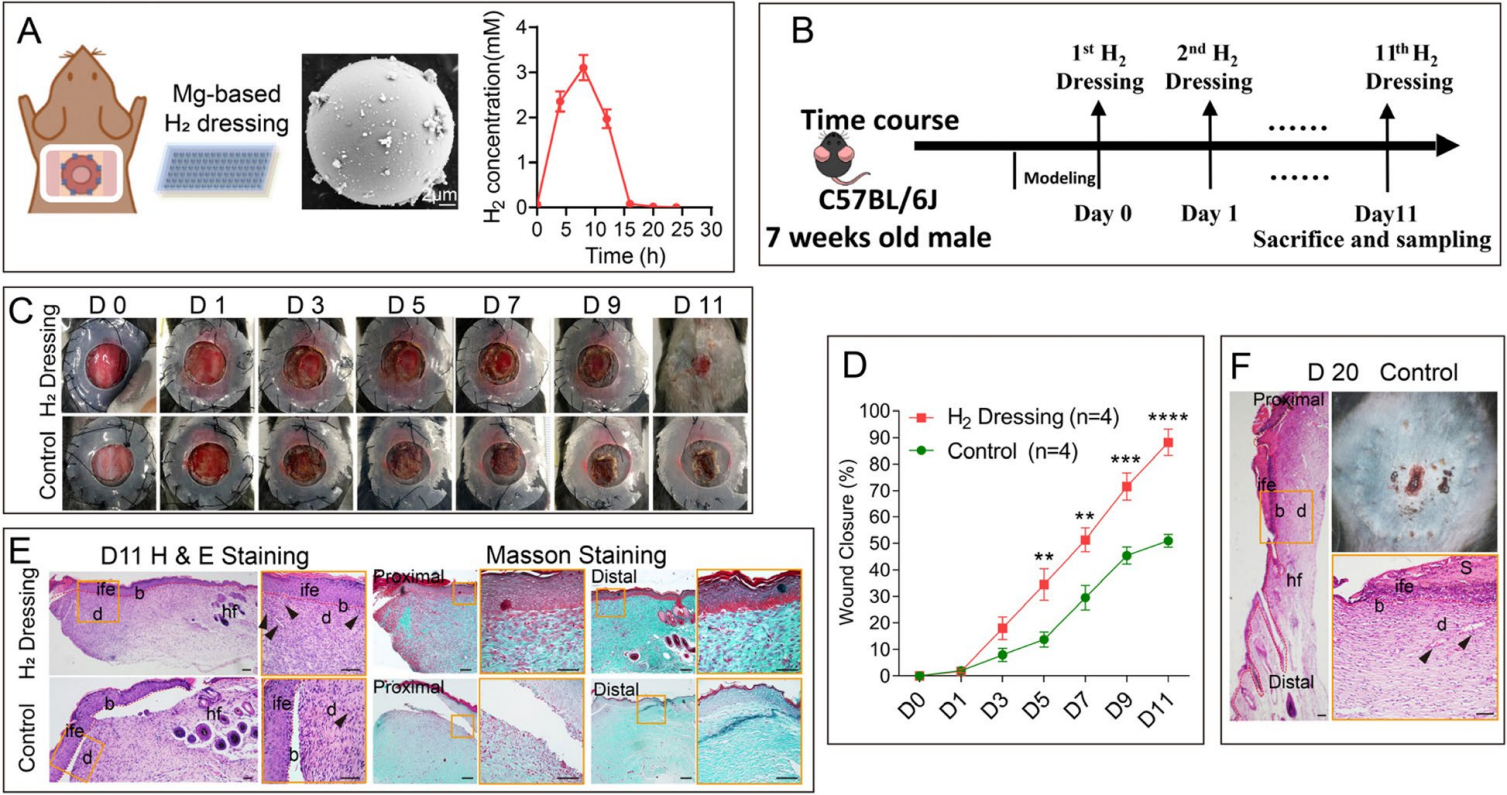
Daily topical treatment of H_2_ dressing significantly promoted cutaneous wound healing. **A** Left: scheme images of Mg-based H_2_ dressing in cutaneous wound healing treatment; Middle: scanning electron microscope (SEM) image of Mg microparticles; right: H_2_ release curves of Mg-based dressing. **B** Timeline of animal experiments and daily H_2_ dressing treatment (or normal dressing condition). **C** Representative wound area images captured every 2 days after modeling. **D** Comparison of wound closure percentage after modeling between H_2_ dressing and control groups. **E**, **F** H&E staining and Masson staining showing the tissue remodeling 11 days and 20 days post-wounding. Data in **D** was analyzed by two-way ANOVA test. All of the data are plotted as mean ± SEM. *P value < 0.05; **P value < 0.01; ***P value < 0.001; ****P value < 0.0001; no stars for *P* value > 0.05; scale bar in A = 2 μm, scale bar in E = 100 μm. Red dotted line in **E** (H&E staining) indicates the boundary between the epithelium and dermis. Black arrowheads in **E** and **F** indicate blood capillaries in the dermal layer of the wound. b, basal layer; d, dermis; hf, hair follicles; ife, interfollicular epithelium; s, scab

Considering the application in human wound care, topical H_2_-releasing dressing could be a candidate strategy which is more convenient and easier to perform. As it is shown in Fig. 6A (left), Mg-based H_2_-releasing dressing is prepared and topical treated on top of the wound. The Mg balls are all microparticles with the average dynamiter of 20 μm (Fig. 6A, middle). The H_2_ release lasted for more than 12 h (Fig. 6A, right). H_2_ dressing group healed faster than the control group (Fig. 6C, D), with more blood capillaries formation and collagen deposition on day 11 post-wounding (Fig. 6E). The day 20 HE staining in the control group (Fig. 6F) suggested that, even though given doubled time the wound closure in the control group reached a same level as the H_2_ group on day 11 post-wounding, the healing effects might be not as good as the H_2_ group, because of the incomplete epithelial layer, unclear basal layer boundary, and less capillaries formation. Additional research into longer-lasting and more thorough healing benefits for hydrogen dressing is still needed, such as in scar formation and tissue remodeling.

## Discussion

### Evaluation of the H_2_ induced novel wound healing pattern

The speed and effect of re-epithelialization during wound healing is largely attributable to ECM [12] and EpSCs [11]. Therefore, it is vital and uplifting to find new strategies that induce autologous ECM accumulation and EpSCs activation for wound healing. In addition to these two factors above, “TIME” is usually being applied in the evaluation of healing effect. Our current study discovered that molecular hydrogen induced a new healing pattern, particularly characterized by fast re-epithelialization, early ECM deposition and EpSCs activation. For the evaluation of the “TIME” indicators, H_2_ promoted the effect of wound healing in a comprehensive way. “T” for tissue remodeling is improved by good DEJ, massive blood vessel formation and dermal collagens deposition. In the “I” of inflammation, we proved that H_2_ reduced the splenic Th_1_ and Th_17_ Th-cells subgroup distribution. This shift of Th-cells subgroup is reported to be important in facilitating wound healing [66]. For “M” of moisture balance, we revealed that H_2_ induced a natural moist-like healing mode, with less blood clots formation. “E” is representing of edge of wound, and H_2_ greatly promoted the re-epithelialization progress, with fast keratinocytes migration and early EpSCs proliferation in the leading edge. However, it has to be further investigated, if our perspective can be applied to all wounds. The animal model of sterile wound model can be one of the limitations in our study. Although there are evidences showing the positive role of hydrogen in multiple wound care, the therapeutic impact and our concept in the mechanism of hydrogen in the treatment of complicated wounds, still need to be further established. Because wounds such as in diabetic wounds and burns, are frequent in clinical practice.

### Early deposition of ECM components promoted by H_2_ and contributed to fast re-epithelialization, tight DEJ and natural moist healing environment

As the most abundant protein in the ECM [67], collagen (especially type I and type III collagen) synthesis, deposition, and release are favourable for EpSCs activities in the skin. In our study, we observed early ECM deposition (1–3 days after wounding) especially for collagens after H_2_ treatment (Fig. 2C–H). This was confirmed by RNA-seq (Figure S3E, F) and untargeted metabolome analysis (Fig. 2K). Though in some hard-to-heal wound studies, H_2_ was proved to promote collagen synthesis by masson staining [40] and IHC staining for Col-1 [42], our study is the first comprehensive and histological reveal of H_2_-induced early deposition of massive ECM components during re-epithelialization of wound healing. In order to re-form the basement membrane beneath the epidermal basal layer, both keratinocytes and fibroblasts will contribute to ECM components synthesizing [12]. Early dermal col-I and especially epidermal col-III in the proximal wound were found accumulated in the H_2_ group, which indicated better structural integrity of the ECM and refined tissue functions [68]. This is possibly related with the transformation from keratinocytes to myoepithelial-like cells (K14^+^/αSMA^+^, Fig. 3A, G). In skin tissue, the ratio of col-I/col-III normally increased with time from foetus to adult [69, 70]. The cross-linking pattern of collagen is always important to determine the scar-free wound [69–72]: foetal skin tissue deposit more col-III than adult skin, which contributes to scar-less wound healing; on the contrary, fibrillar col-I is dominant in adult skin, leading to scar formation. During wound healing, col-I/col-III ratio stayed dynamically, first decreased and then increased to normal level [72]. Therefore, the massive deposition of col-III in the proximal wound (Fig. 2D) after H_2_ treatment may indicate a youthful state of skin, leading to faster re-epithelialisation. In addition to col-I and col-III, H_2_ induced early fibronectin deposition would also provide a scaffolding for epithelial migration [73], which is similar to fetal scarless wound healing pattern [74]. Skin basal keratinocytes separate the epidermis and dermis, and maintain the two layers attached together. This function is dependent on DEJ-related proteins, of which Laminins [75] and col-XVII [76] are two of the most important members. The higher expression level of laminin (Fig. 2H) and especially col-XVII (Fig. 2G) in the proximal epidermal keratinocytes indicated better DEJ induced by H_2_. Provision and maintenance of a moist condition (Fig. 2J) is favourable for establishing an ideal microenvironment for healing processes. Collagen dressing is therefore always chosen as one of the wound care strategies [77, 78]. Instead of topical application of collagens, early autologous collagens accumulation in the proximal dermal and epidermal wound was found induced by H_2_ and persisted in the whole healing processes. Untargeted metabolome analysis for the wound bed tissue revealed that most of the identified differential metabolites in H_2_ group (Fig. 2K) are related with the components, precursors, or inducers of collagen synthesis, such as 4-hydroxyproline [79], L-proline [80], and asymmetric dimethylarginine [81]. Taken together, ECM deposition promoted by H_2_ could contribute to the fast re-epithelialization, tight DEJ, and natural moist healing environment.

### The early activation of autologous EpSCs proliferation and differentiation regulated by H_2_ treatment

Once the cells at the wound edge begin to migrate, epithelial cells behind the edge proliferate; this continues until new epithelium covers the damaged tissue [82]. Epidermal growth and thickening rely on the surrounding components such as the ECM, as well as on the activation of EpSCs. To restore the functional epidermal barrier, EpSCs proliferation is followed by differentiation [83, 84]. When IFESCs and HFSCs are recruited to the IFE after injury, they progressively lose their initial identities and differentiate [85]. Different stem cell markers involved in IFESC and HFSCs niches [86], such as K5 [87], Lgr6 [88], K15 [89], and p63 [90] were examined here. We observed early proliferation (since day 1 post-wounding, about 1–2 days earlier than the control group) of IFESCs and HFSCs during the first 3 days post-wounding (Fig. 4A, B, G–I), as well as thickening of proximal stratum corneum in the H_2_ group (Fig. 2A), though the differentiation trajectory of these stem cells still needs to be tracked. Two other stem cell–related studies reported that H_2_ prolonged the replicative lifespan of bone marrow multipotential stromal cells in vitro [46], and protected hematopoietic stem cell from irradiation injury [47]. In vivo studies on the effect of hydrogen on stem cell proliferation and differentiation, and the relationship between hydrogen and EpSCs in wound healing have not been studied. Our study revealed that H_2_ promoted the early EpSCs proliferation and differentiation in different time series (day 1–3) and space series (leading edge, mid-end and distal part of wound) during wound healing for the first time. The following differentiation of the EpSCs then surprisingly turned out to be K14^+^/α-SMA^+^ myoepithelial-like cells, which may further contribute to collagen deposition, epidermis migration and the maintenance of the youthful state of cells. In addition, some studies have already mentioned the mobilization and contribution of MSCs/stroma cells to regeneration of injured epithelia [91]. In our study, in vitro experiment also proved that H_2_ could activate MSCs and induce the transformation from MSCs towards myofibroblast-like direction, with the phenotypes of αSMA^+^, Vemintin^+^, and better ability of collagen producing (Figure S7) and moving (Fig. 5G, H), which could further contribute to the wound closure. The combined major benefits of earlier ECM deposition and stem cell proliferation and differentiation after H_2_ treatment, as well as vessel formation and cell migration, promoted haemostasis, re-epithelialization, and reduced scab formation. However, whether EpSCs in a collagen-rich environment are more likely to differentiate to keratinocytes remains unclear [92]. Besides, in order to identify which EpSCs subgroups were first triggered by H_2_ and their subsequent differentiation trajectory throughout wound healing, more studies on the EpSCs linage tracking are still needed.

### Potential mechanism of H_2_-induced high cellular activity in various cells

Notably, though most of the current reports have focused on H_2_’s anti-ROS and anti-oxidative functions [93], our study revealed that these anti-oxidative functions are not the key means by which H_2_ (O_2_-indenpendent) accelerates wound healing. One direct evidence could be NAC, which is well-known as an effective ROS scavenger [94] , had no statistical effect on the healing processes in our study (Fig. 1C, D). Meanwhile, the healing effects in 33% O_2_ treatment had no significant difference comparing with the control group (Fig. 1C, D). Consequently, the mechanism of selective antioxidant cannot explain the H_2_ induced early accumulation of ECM and activation of EpSCs observed in our study, and that, these H_2_-induced beneficial effects in wound healing is not O_2_ dependent. Besides, we discovered that H_2_ promoted cell viability in vitro of different cell types, which manifested in more tube formation for endothelial cells (Fig. 1N), faster cell migration for fibroblasts (Fig. 5E, G, I), and better epithelialization for keratinocytes (Fig. 5A). This is also reflected in EpSCs early proliferation, however, the proliferation seemed to be under a strict regulation, with proliferation during the first 2 days post-wounding (Fig. 4A, B, G–I) and differentiation from day 3 onward (Fig. 3A, G). The promoted cell viability was quite consistent with what we observed in the H_2_ group in vivo, including early blood vessel formation (visible since day 3 post-wounding, Fig. 1L, M), fibroblast aggregation (approximately day 3 post-wounding, Fig. 3J, L) in the proximal wound and faster re-epithelialization (visible since day 3 post-wounding, Fig. 2A). These observations above revealed that H_2_ was able to boost the cell viabilities of diverse cells in different ways, at different time points but in the same histological space during wound healing. Combining with our results (Fig. 5C, D, K–N; Figure S3H), one of the possible explanations for the better cell viability could be H_2_ induce robust mitochondrial activities and less structurally aberrant mitochondria during wound healing. This will further promote such as cell proliferation [95] and migration [96] abilities. Though the mechanism of H_2_ remains unclear, recent studies revealed that H_2_ may be multi-targeted and mainly based on enzymatic reactions. Higher organisms may have hydrogen metabolism abilities based on hydrogenase, especially for the mitochondrial-related hydrogen metabolism [97, 98]. The cell membrane may have the enzyme activity of hydrogen metabolism, and ion channels are also possibly regulated by H_2_ [99, 100]. Recent study also put forward that, Fe-porphyrin is a H_2_-targeted molecule, acting as a biosensor and catalyst for H_2_ [101, 102]. Taken together, our study indicates that the role of H_2_ may function at the level of very basic biological processes of life, which needs to be further explored in the future.

## Conclusion

**Fig. 7:**
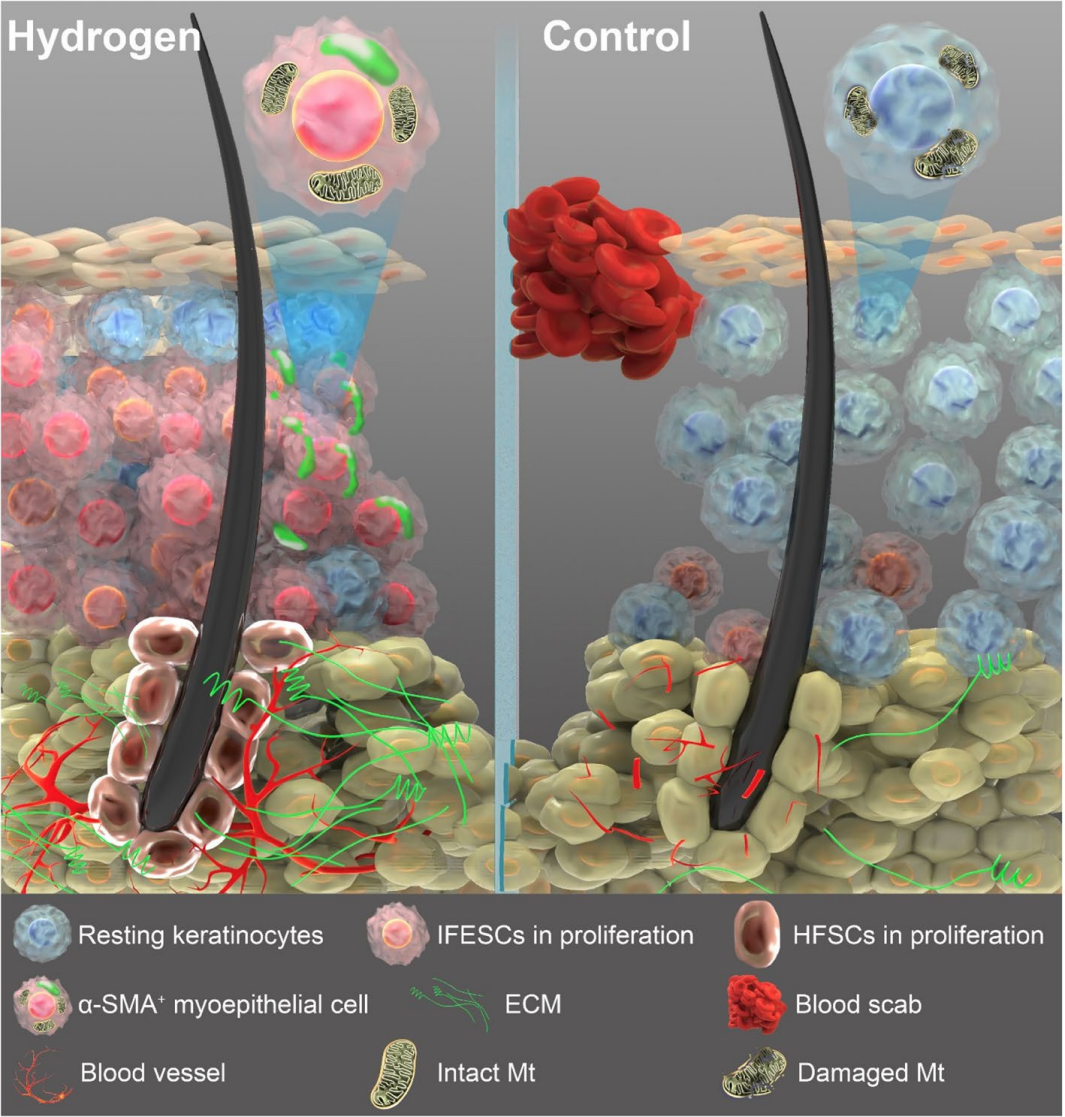
Schematic representation of the skin under H_2_ treatment and normal conditions during the early stage wound healing. In addition to anti-inflammation (especially for Th1- and Th17-related systemic inflammation), H_2_ treatment brings multiple other benefits (shown in the figure) including faster epidermis thickening, earlier proliferation (red nuclei) of epidermal stem cells (basal cells and hair follicle cells), early differentiation of basal cells in the wound edge (differentiate towards α-SMA^+^ cell type), earlier and better ECM deposit, faster blood vessel formation, mitochondrial damage repair, and moist healing process (less blood scab formation)

For this study, we used a H_2_ chamber (no requirement for body fixation or anaesthesia) with a murine aseptic wound model. Considering its both inhalation and wound surface contact with H_2_, we also put efforts in the nasal inhalation (chronic wound) [103] and topical H_2_ sustained-release dressings (cutaneous wound) (Fig. 6) treatment in wound care, and both non-invasive interventions received good effects. Clinical investigations and other wound models with more complexity are also being conducted. There is also evidences that topical hydrogen intervention improved the wound healing in vivo [104]; however, comparing to focusing on the anti-infection and anti-inflammation effects, our research, instead, have indicated more functions of H_2_ in wound healing. Without combination with any specific medicine, we revealed that H_2_ alone was able to increase the rate of wound healing, with advantages (Fig. 7) including but not limited to promoting ECM deposition, autonomous stem cell early proliferation, blood vessel formation, cell viability, and natural moist healing pattern. We believe that this highly effective therapeutic method of H_2_ treatment holds promise for further clinical wound treatment, as well as applications in the tissue damage repair, regeneration and other wider fields.

## Supplementary Information

**Additional file 1: Supplemental Figure S1.** H_2_ slightly increases growth factors concentration at wound sites at day 1-3 after wounding. A, B. Profiles of tissue growth factors PDGF and EGF among the three groups at time points of day 1, day 2 and day 3 post-wounding. C, D. Concentrations of tissue growth factors bFGF and TGFβ-1 among all three groups at day 3. Data in A and B processed Two-way ANOVA test, and data in C and D processed unpaired t test. All data were plotted as Mean±SEM. * *P*-value < 0.05; ** *P*-value <0.01; *** *P*-value < 0.001; no stars for *P*-value > 0.05. **Supplemental Figure S2.** Whole-mount scanning showing H_2_ promoted early tube formation at the first 2 days after wounding. A and B. Panoramic scanning of wound edge and the representative immunofluorescence images of CD31 (green) and k14 (red) expression in the leading edge (L, 0-1 mm from wound edge), mid-end (M, 1-2 mm from wound edge) and distal (D, 2-3 mm from wound edge) of the wound at day 1 and day 2 post wounding respectively. White dotted line indicates the boundary between the epithelium and dermis. White arrowhead indicates tube formation. Scale bar = 100 μM. **Supplemental Figure S3.** Whole-mount scanning showing H_2_ promoted early tube formation at the first 2 days after wounding. **A** and **B**. Representative microscopic images and the quantification of in vitro blood vessel formation of Human Umbilical Vein Endothelial Cells (HUVEC) at 12 h after different treatments. Red hatched line outlines the newly formed tubes. Data in **B** were processed unpaired multiple T test. All of the data are plotted as Mean±SEM. * *P*-value < 0.05; ** *P*-value <0.01; *** *P*-value < 0.001; no stars for *P*-value > 0.05. Scale bar = 100 μM. **Supplemental Figure S4.** Comprehensive gene set function enrichment analysis of differential gene expression induced by 66% H_2_ treatment at the first 3 days post wounding. A. Heat map showing the differentially expressed genes (DEGs) among the three time points (D1H vs D1C, D2H vs D2C, D3H vs D3C). B. Venn diagrams showing the overlapping number of DEGs among the comparative data of three time points. C. Counting of total, up- and downregulated DEGs among the comparative data of three time points. D. Heat map showing four clusters identified from all the DEGs. E. GO-BP analysis in each individual cluster, the GO terms for genes related with extracellular matrix organization and muscle cell differentiation were significantly enriched in D1H and D3H, separately. F. Top 10 enriched up and down GO-BP in D1H by GSEA. G. Top 10 enriched up and down GO-BP in D2H by GSEA. H. Top 10 enriched up and down GO-BP in D1H by GSEA. **Supplemental Figure S5.** Transcriptomics and metabolomics correlation analysis reveal that differential genes and metabolites of the D3H group are enriched in tight junction and focal adhesion pathways. A. Correlation plot of DEGs and differential metabolites between D3H and D3C. B. Count of D3H vs D3C DEGs enriched in KEGG pathways. **Supplemental Figure S6.** ECM components col-I, fibronectin, laminin, and integrin deposition 2 days after wounding are increased in the H_2_ group especially in the proximal wound edge. IHC staining showing the difference between D2H and D2C in ECM deposit of day 2 post wounding. A-D indicated Col-1, fibronectin, laminin and integrin expression in D2H and D2H, separately. Scale bar = 100 μm. **Supplemental Figure S7.** H_2_ conditioned medium promotes collagen deposition *in vitro* in MSCs, fibroblasts and keratinocytes. **A.** Three positive markers in MSCs phenotype. **B.** Five negative markers in MSCs phenotype. **C**. α-SMA, Vimintin, and Col-1 expression in the HUCPF (fibroblast) between 66% H_2_ and control groups 24 h after treatment. **D** and **E**. Col-1 deposit in the HaCat (keratinocyte) and HPF (fibroblast) cells between 66% H_2_ and control groups 24 h after treatment. **Supplemental Figure S8.** 24-, 48-, and 72-h time points tissue cytokine profiling and four-color Flow CytoMetry (FCM) of different targets showing quantification of CD3^+^, CD4^+^, and CD8^+^ T cells, as well as of Th_1_, Th_2_, Th_17_ and Treg subgroups. A. FCM quantification of CD4^+^ and CD8^+^ cell distribution in all three groups. B. T-bet^+^/CD4^+^ T-cell distribution, double checked against IFN-γ^+^/CD4^+^ T-cell distribution. C. GATA3^+^/CD4^+^ T-cell distribution, double-checked against IL-4^+^/CD4^+^ T-cell distribution. D. IL-17A^+^/CD4^+^ T-cell distribution. E. CD25^high^/CD127^low^ T-cell distribution. F. Example figures of gating strategy used in cytometry flow test, and the first four plots showed procedure of gating strategy from one of the typical samples in Th_1_ subgroup cells analysis, the form showed the cell abundance during the gating strategy. G. Pro-inflammatory cytokine profile (TNF-α, IL-1β, and IL-6), Th_17_-related cytokine (IL-17a and IFN-γ), and Anti-inflammatory cytokine IL-10 expression profile in all of the three groups at three time points. Data in A-C and G processed Two-way ANOVA test, and data in D and E processed unpaired t test. All data were plotted as Mean±SEM. * *P*-value < 0.05; ** *P*-value <0.01; *** *P*-value < 0.001; no stars for *P*-value > 0.05. **Supplemental Figure S9.** High concentration of H_2_ promoted early mussel and nerve repair. A. Left: Scheme of a mouse gastrocnemius strike model. Right: Timeline of animal experiments and daily H_2_ treatment. B. Capture of the gastrocnemius strike area and blood perfusion 0-72 hours post-wounding in the 66% H_2_ Control groups. C. & D. Quantification of diameter and blood perfusion of the gastrocnemius strike area between two groups. E. & F. Representative immunofluorescence images for NFH (green) nerve injury repair and axons maturation in dermal wounds of at day 3 and 11 post wounding. Data in C & D were processed unpaired T test. All of the data are plotted as Mean±SEM. * *P*-value < 0.05; ** *P*-value <0.01; *** *P*-value < 0.001; no stars for *P*-value > 0.05; Scale bar = 100 μm. White dotted line in E & F indicates the boundary between the epithelium and dermis. Arrow in E & F indicates NFH^+^ cells. **Table S1.** List of primary and secondary antibodies used in experiments. IHC indicates immunochemistry, IF indicates immunofluorescence; FC indicates flow cytometry. **Table S2.** Gradient conditions for reversed phase C18 separation. **Table S3.** Gradient conditions for HILIC separation of polar metabolites. **Table S4.** DEGs annotation of all the 6 groups. **Table S5.** DEGs annotation of D1H vs D1C. **Table S6.** DEGs annotation of D2H vs D2C. **Table S7.** DEGs annotation of D3H vs D3C. **Table S8.** Raw data of nontargeted metabolomic analysis between D3H and D3C. **Table S9.** 12 metabolites identified through hydrophilic-product test between 66% H2 and Control group. **Table S10.** Top 20 significant pathways discovered by nontargeted metabolomic analysis between the D3H and D3C groups.

**Additional file 2: Movie S1.** Live cell imaging of HPF cells under H_2_ medium condition within 24 h. HPF cells stained by actin-GFP images were taken every one hour, and 0, 4, 8, 12, 16, 20 and 24 h images were collected to build the video. Bar indicates 1000 μ.

**Additional file 3: Movie S2.** Live cell imaging of HPF cells under normal condition within 24 h. HPF cells stained by actin-GFP images were taken every one hour, and 0, 4, 8, 12, 16, 20 and 24 h images were collected to build the video. Bar indicates 1000 μM.

## Data Availability

The datasets used and/or analyzed during the current study are available from the corresponding author on reasonable request.

## Abbreviations

DEJ: Dermal and epidermal junction
DEGs: Differentially expressed genes
DSP: Digital spatial profiler
ECM: Extracellular matrix
EpSCs: Epidermal stem cells
GO-BP: Gene Ontology biological process
GSEA: Gene set enrichment analysis
HFs: Hair follicles
H&E: Hematoxylin and eosin
HUCP-MSCs: Human umbilical cord primary mesenchymal stem cells
IFE: Interfollicular epidermis
IHC: Immunohistochemical staining
Mt: Mitochondria
NAC: N-acetyl-L-cysteine
ROS: Reactive oxygen species
