# Supplementary material for: Molecular hydrogen promotes wound healing by inducing early epidermal stem cell proliferation and extracellular matrix deposition
Source: Inflamm Regen. 2023 Mar 28;43:22. doi: 10.1186/s41232-023-00271-9 (PMC10044764; doi:10.1186/s41232-023-00271-9)
Supplement: Supplementary file 1 — Additional file 1: Supplemental Figure S1. H2 slightly increases growth factors concentration at wound sites at day 1-3 after wounding. A, B. Profiles of tissue growth factors PDGF and EGF among the three groups at time points of day 1, day 2 and day 3 post-wounding. C, D. Concentrations of tissue growth factors bFGF and TGFβ-1 among all three groups at day 3. Data in A and B processed Two-way ANOVA test, and data in C and D processed unpaired t test. All data were plotted as Mean±SEM. * P-value < 0.05; ** P-value <0.01; *** P-value < 0.001; no stars for P-value > 0.05. Supplemental Figure S2. Whole-mount scanning showing H2 promoted early tube formation at the first 2 days after wounding. A and B. Panoramic scanning of wound edge and the representative immunofluorescence images of CD31 (green) and k14 (red) expression in the leading edge (L, 0-1 mm from wound edge), mid-end (M, 1-2 mm from wound edge) and distal (D, 2-3 mm from wound edge) of the wound at day 1 and day 2 post wounding respectively. White dotted line indicates the boundary between the epithelium and dermis. White arrowhead indicates tube formation. Scale bar = 100 μM. Supplemental Figure S3. Whole-mount scanning showing H2 promoted early tube formation at the first 2 days after wounding. A and B. Representative microscopic images and the quantification of in vitro blood vessel formation of Human Umbilical Vein Endothelial Cells (HUVEC) at 12 h after different treatments. Red hatched line outlines the newly formed tubes. Data in B were processed unpaired multiple T test. All of the data are plotted as Mean±SEM. * P-value < 0.05; ** P-value <0.01; *** P-value < 0.001; no stars for P-value > 0.05. Scale bar = 100 μM. Supplemental Figure S4. Comprehensive gene set function enrichment analysis of differential gene expression induced by 66% H2 treatment at the first 3 days post wounding. A. Heat map showing the differentially expressed genes (DEGs) among the three time points (D1H vs D1C, D2H vs D2C, D3H [file 41232_2023_271_MOESM1_ESM.zip › Supplemental information- 3rd submission.docx]

**Supplemental Information**

**Molecular hydrogen promotes wound healing by inducing early epidermal stem cell proliferation and extracellular matrix deposition**

Pengxiang Zhao^1,2,3^, Zheng Dang^1,2,3^, Mengyu Liu^1,2,3^, Dazhi Guo^4^, Ruiliu Luo^1,2,3^, Mingzi Zhang^5^, Fei Xie^1,2,3^, Xujuan Zhang^1,2,3^, Youbin Wang^5^, Shuyi Pan^4^, and Xuemei Ma^1,2,3^*

**Affiliations:**

^1^ Faculty of Environment and Life Science, Beijing University of Technology, Beijing 100124, P. R. China;

^2^ Beijing molecular hydrogen research center, Beijing 100124, P. R. China;

^3^ Beijing International Science and Technology Cooperation Base of Antivirus Drug, Beijing 100124, P. R. China;

^4^ Department of Hyperbaric Oxygen, Sixth Medical Center of PLA General Hospital, Beijing 100048, P. R. China;

^5^ Department of Plastic Surgery, Peking Union Medical College Hospital (Dongdan campus), No.1 Shuaifuyuan Wangfujing Dongcheng District, Beijing 100730, P.R. China.

*To whom correspondence should be addressed: [xmma@bjut.edu.cn](mailto:xmma@bjut.edu.cn), Telephone number: +8613661188049.

**
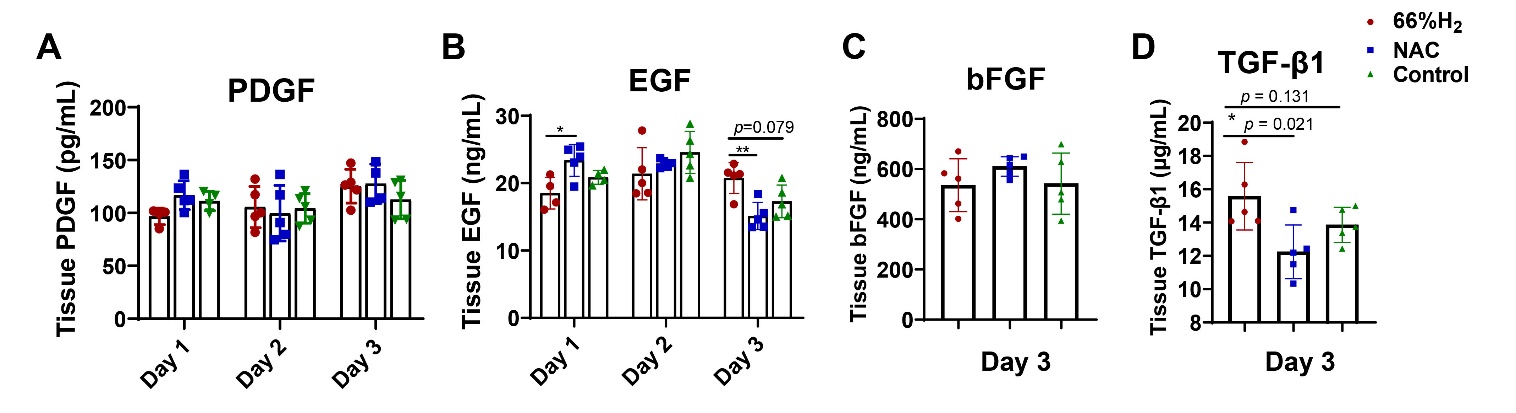
**

**Supplemental Figure 1. H_2_ slightly increases growth factors concentration at wound sites at day 1-3 after wounding.**

**A**, **B**. Profiles of tissue growth factors PDGF and EGF among the three groups at time points of day 1, day 2 and day 3 post-wounding. **C**, **D**. Concentrations of tissue growth factors bFGF and TGFβ-1 among all three groups at day 3. Data in A and B processed Two-way ANOVA test, and data in **C** and **D** processed unpaired t test. All data were plotted as Mean±SEM. * *P*-value < 0.05; ** *P*-value <0.01; *** *P*-value < 0.001; no stars for *P*-value > 0.05

**
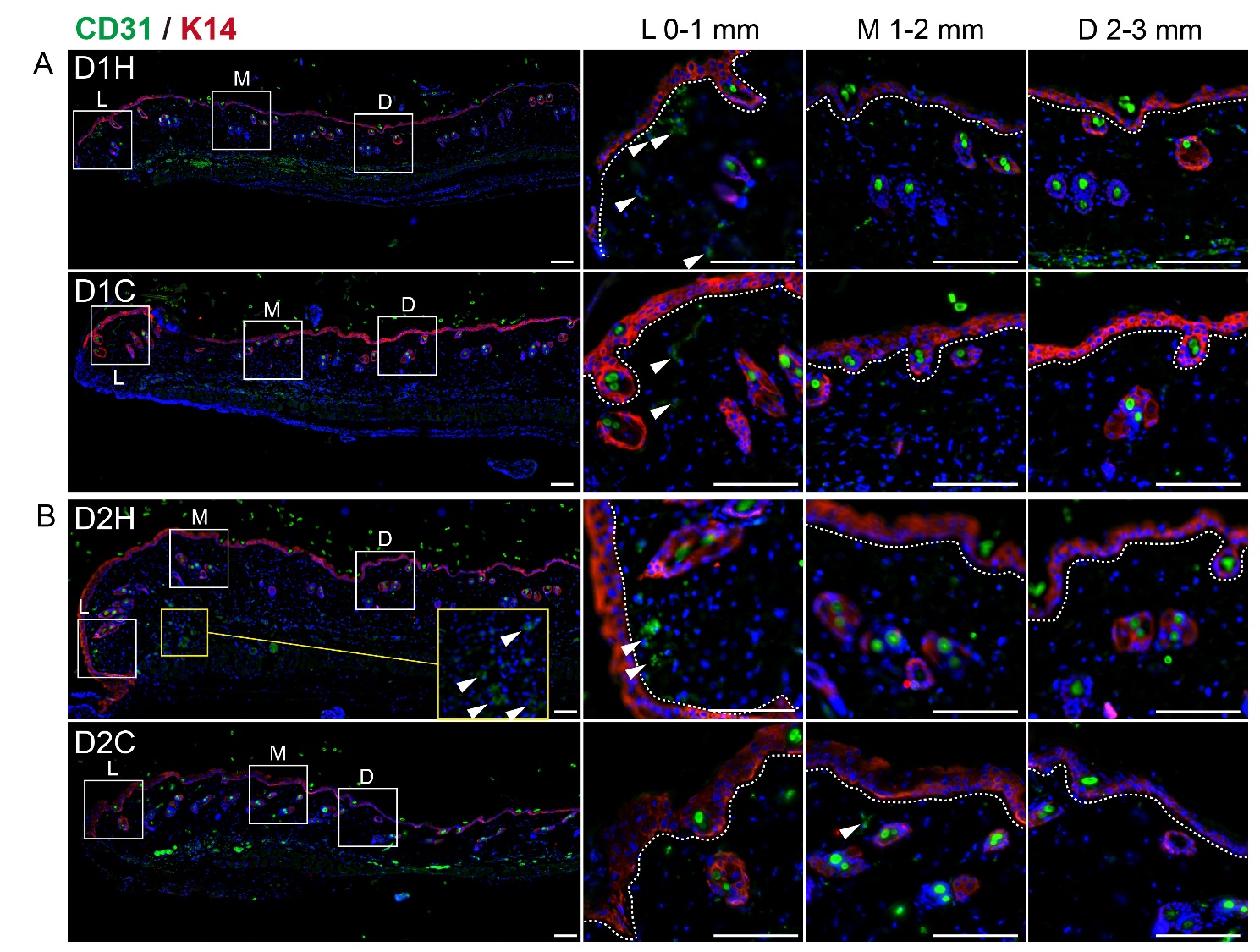
**

**Supplemental Figure 2.** **Whole-mount scanning showing H_2_ promoted early tube formation at the first 2 days after wounding.**

**A** and **B**. Panoramic scanning of wound edge and the representative immunoﬂuorescence images of CD31 (green) and k14 (red) expression in the leading edge (L, 0-1 mm from wound edge), mid-end (M, 1-2 mm from wound edge) and distal (D, 2-3 mm from wound edge) of the wound at day 1 and day 2 post wounding respectively. White dotted line indicates the boundary between the epithelium and dermis. White arrowhead indicates tube formation. Scale bar = 100 μM.

**
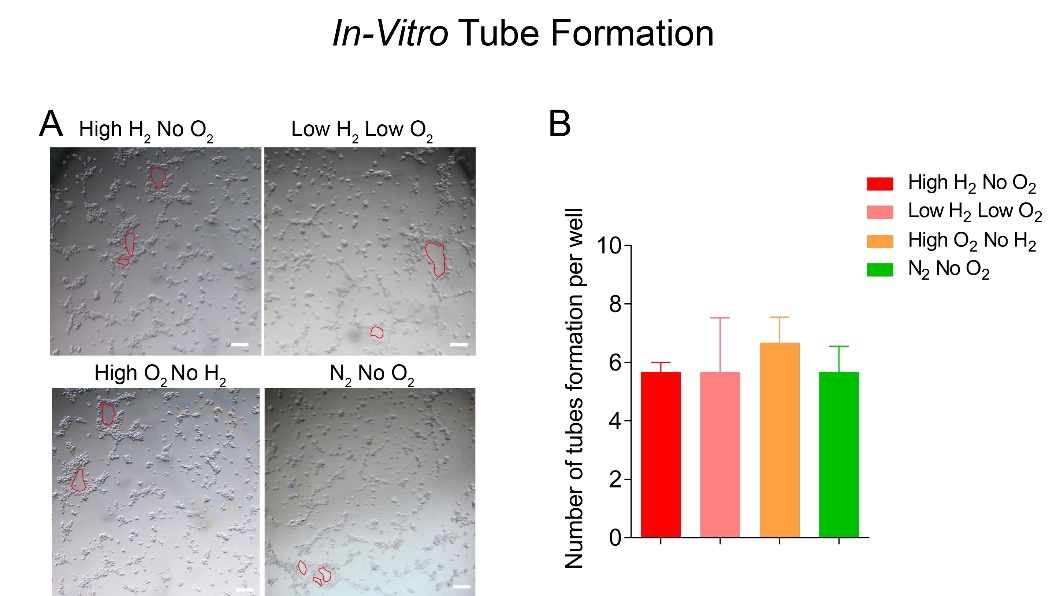
**

**Supplemental Figure 3.** **Whole-mount scanning showing H_2_ promoted early tube formation at the first 2 days after wounding.**

**A** and **B**. Representative microscopic images and the quantification of in vitro blood vessel formation of Human Umbilical Vein Endothelial Cells (HUVEC) at 12 h after different treatments. Red hatched line outlines the newly formed tubes.

Data in **B** were processed unpaired multiple T test. All of the data are plotted as Mean±SEM. * *P*-value < 0.05; ** *P*-value <0.01; *** *P*-value < 0.001; no stars for *P*-value > 0.05. Scale bar = 100 μM.

**
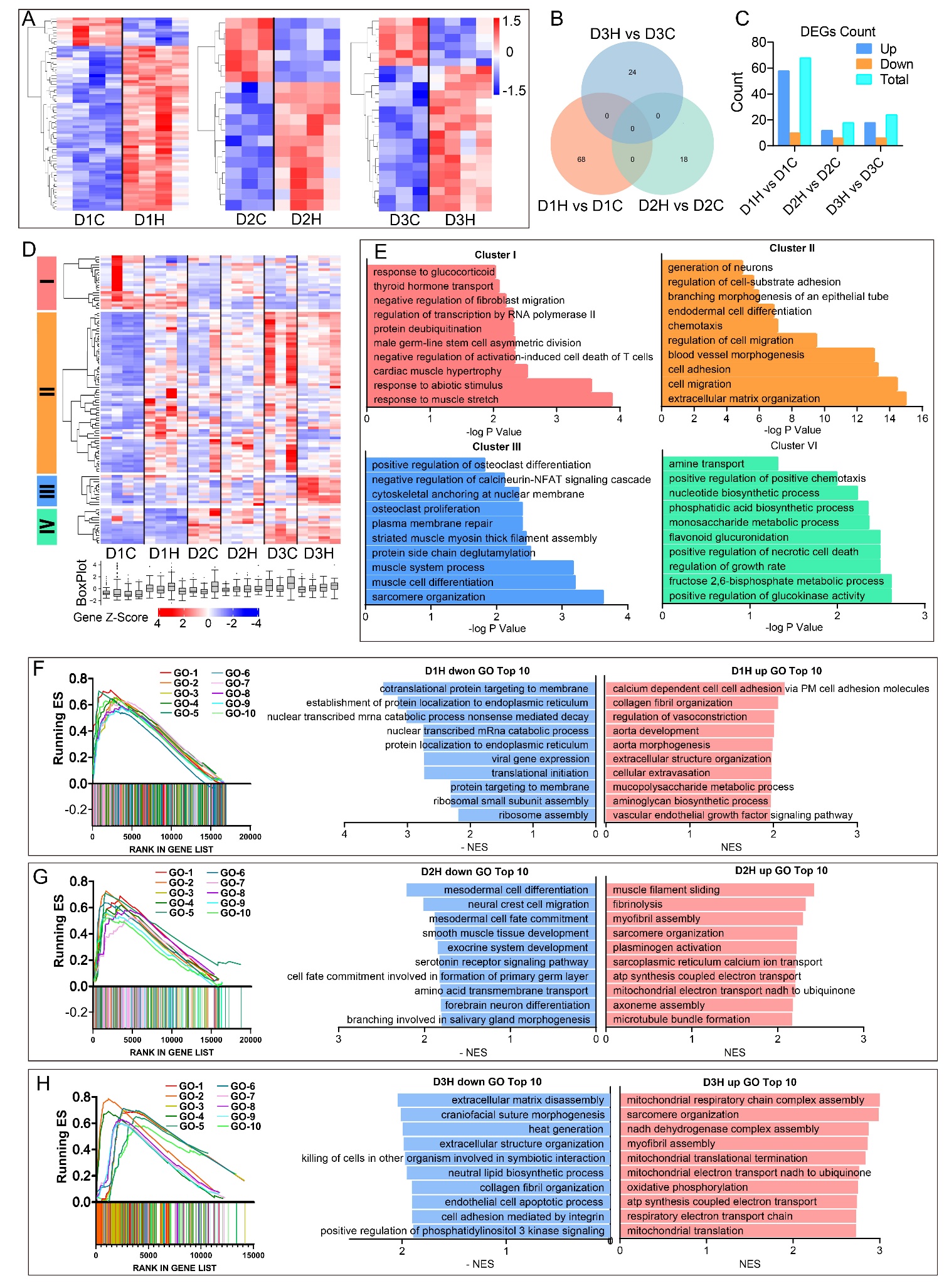
**

**Supplemental Figure 4. Comprehensive gene set function enrichment analysis of differential gene expression induced by 66% H_2_ treatment at the first 3 days post wounding**

**A**. Heat map showing the differentially expressed genes (DEGs) among the three time points (D1H vs D1C, D2H vs D2C, D3H vs D3C). **B**. Venn diagrams showing the overlapping number of DEGs among the comparative data of three time points. **C**. Counting of total, up- and down-regulated DEGs among the comparative data of three time points. **D**. Heat map showing four clusters identified from all the DEGs. **E**. GO-BP analysis in each individual cluster, the GO terms for genes related with extracellular matrix organization and muscle cell differentiation were significantly enriched in D1H and D3H, separately. **F**. Top 10 enriched up and down GO-BP in D1H by GSEA. **G**. Top 10 enriched up and down GO-BP in D2H by GSEA. **H**. Top 10 enriched up and down GO-BP in D1H by GSEA.

**
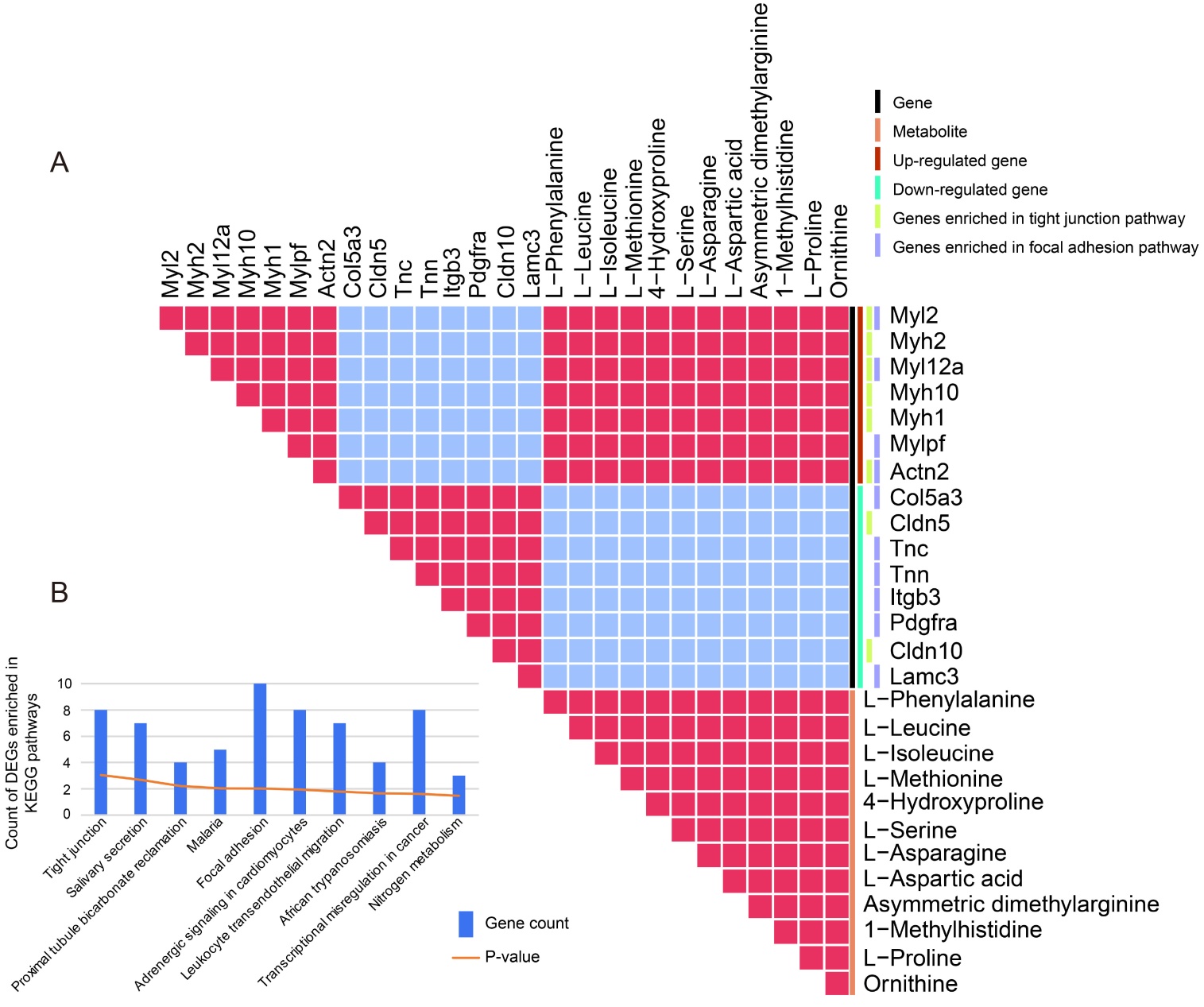
**

**Supplemental Figure 5. Transcriptomics and metabolomics correlation analysis reveal that differential genes and metabolites of the D3H group are enriched in tight junction and focal adhesion pathways.**

A. Correlation plot of DEGs and differential metabolites between D3H and D3C. B. Count of D3H vs D3C DEGs enriched in KEGG pathways.

**
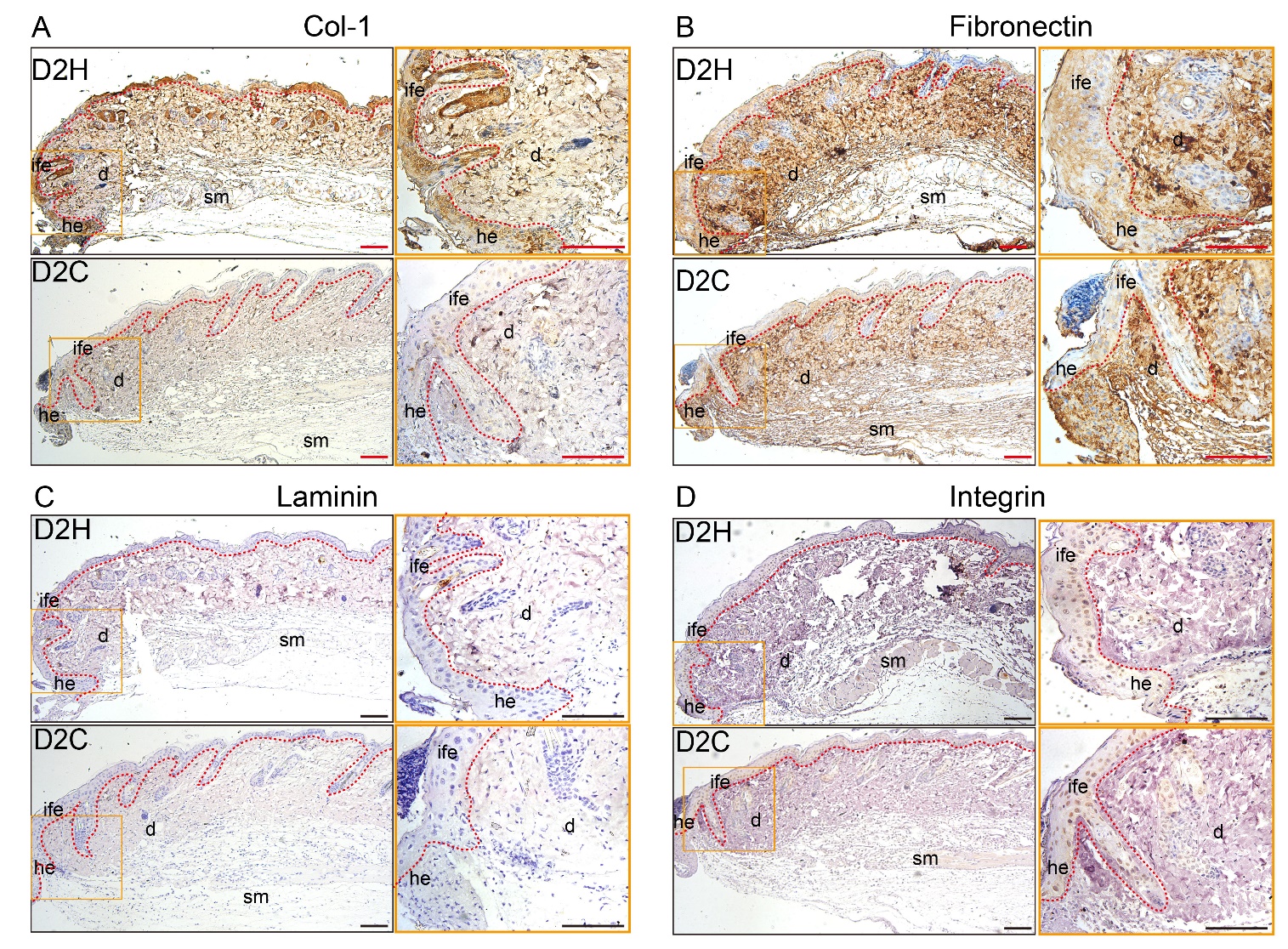
**

**Supplemental Figure 6. ECM components col-I, fibronectin, laminin, and integrin deposition 2 days after wounding are increased in the H_2_ group especially in the proximal wound edge.**

IHC staining showing the difference between D2H and D2C in ECM deposit of day 2 post wounding. **A-D** indicated Col-1, fibronectin, laminin and integrin expression in D2H and D2H, separately. Scale bar = 100 μm.


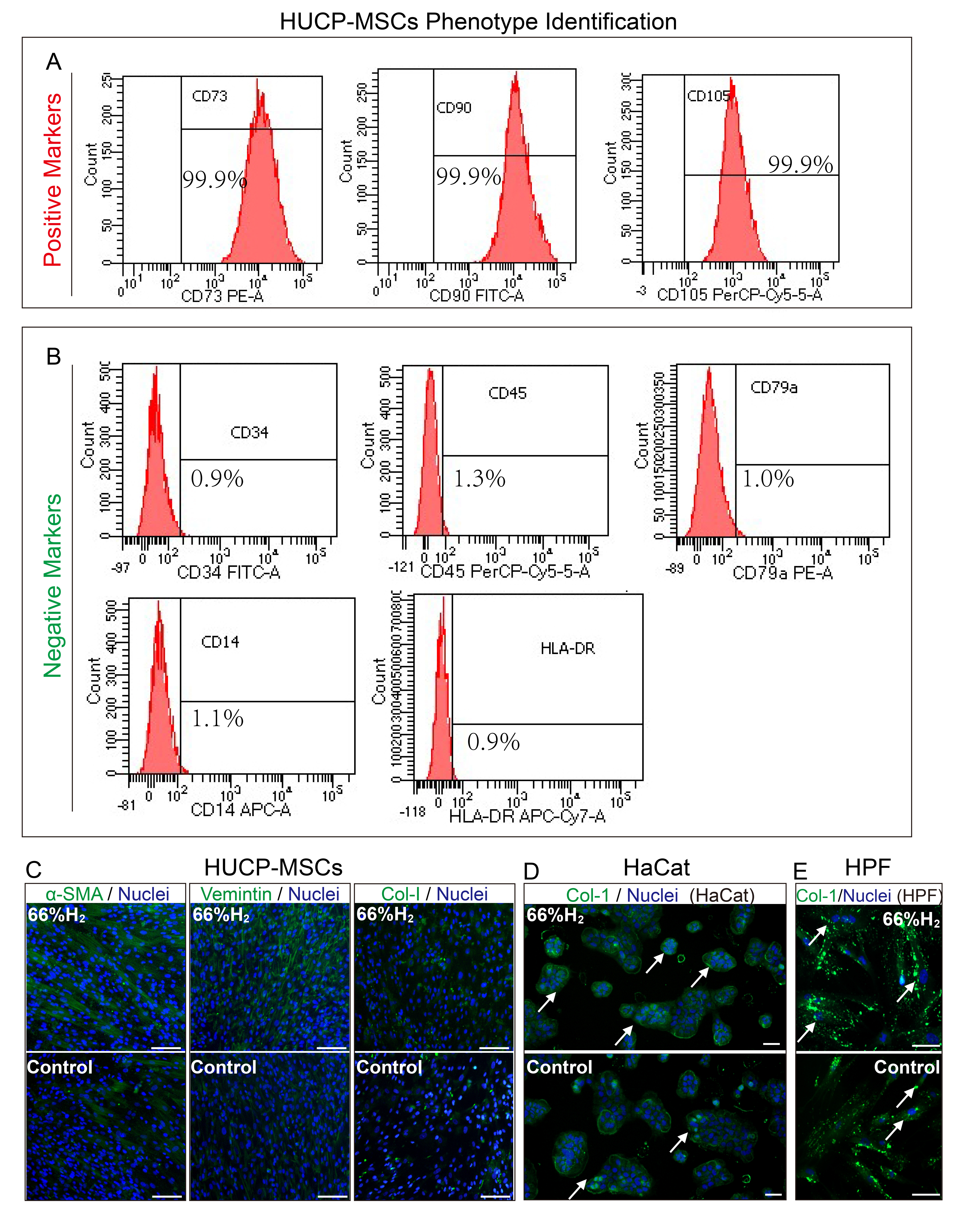


**Supplemental Figure 7. H_2_ conditioned medium promotes collagen deposition *in vitro* in MSCs, fibroblasts and keratinocytes**

**A.** Three positive markers in MSCs phenotype. **B.** Five negative markers in MSCs phenotype. **C**. α-SMA, Vimintin, and Col-1 expression in the HUCPF (fibroblast) between 66% H_2_ and control groups 24 h after treatment. **D** and **E**. Col-1 deposit in the HaCat (keratinocyte) and HPF (fibroblast) cells between 66% H_2_ and control groups 24 h after treatment.

**
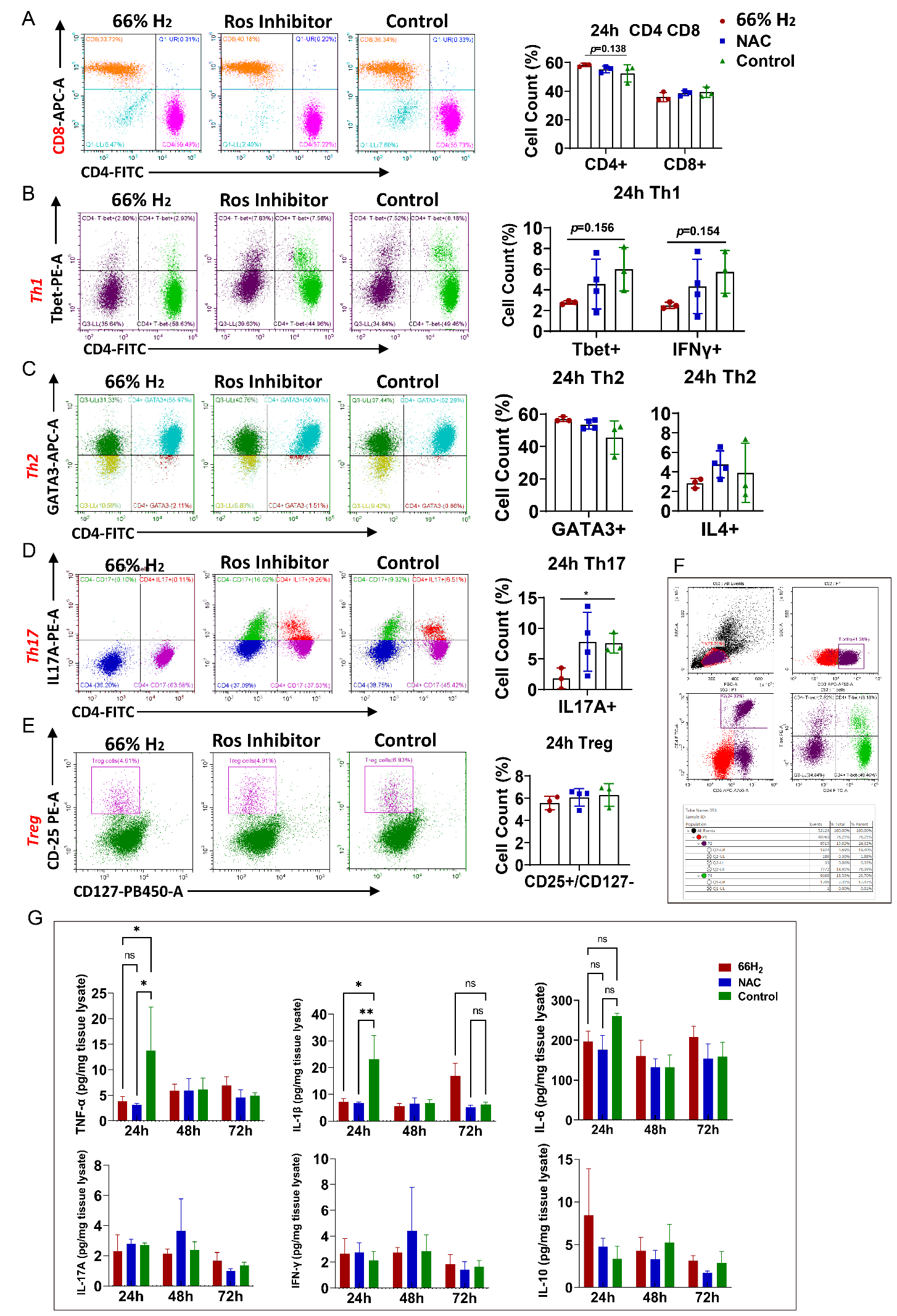
**

**Supplemental Figure 8. 24-, 48-, and 72-h time points tissue cytokine profiling and four-color Flow CytoMetry (FCM) of different targets showing quantification of CD3^+^, CD4^+^, and CD8^+^ T cells, as well as of Th_1_, Th_2_, Th_17_ and Treg subgroups.**

**A**. FCM quantification of CD4^+^ and CD8^+^ cell distribution in all three groups. **B**. T-bet^+^/CD4^+^ T-cell distribution, double checked against IFN-γ^+^/CD4^+^ T-cell distribution. **C**. GATA3^+^/CD4^+^ T-cell distribution, double-checked against IL-4^+^/CD4^+^ T-cell distribution. **D**. IL-17A^+^/CD4^+^ T-cell distribution. **E**. CD25^high^/CD127^low^ T-cell distribution. **F**. Example figures of gating strategy used in cytometry flow test, and the first four plots showed procedure of gating strategy from one of the typical samples in Th_1_ subgroup cells analysis, the form showed the cell abundance during the gating strategy. **G**. Pro-inflammatory cytokine profile (TNF-α, IL-1β, and IL-6), Th_17_-related cytokine (IL-17a and IFN-γ), and Anti-inflammatory cytokine IL-10 expression profile in all of the three groups at three time points. Data in **A-C** and **G** processed Two-way ANOVA test, and data in **D** and **E** processed unpaired t test. All data were plotted as Mean±SEM. * *P*-value < 0.05; ** *P*-value <0.01; *** *P*-value < 0.001; no stars for *P*-value > 0.05.


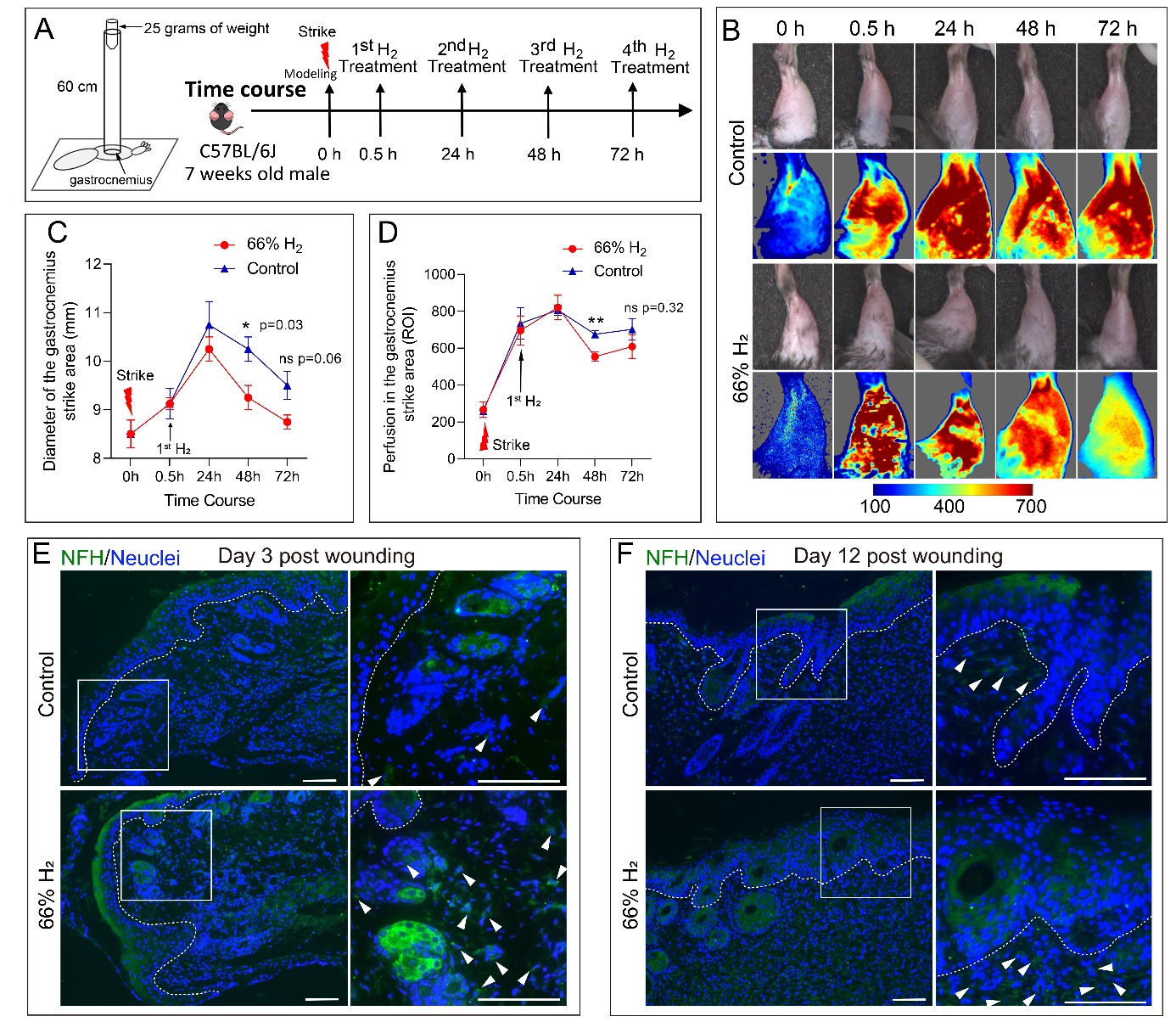


**Supplemental Figure 9. High concentration of H_2_ promoted early mussel and nerve repair.**

**A**. Left: Scheme of a mouse gastrocnemius strike model. Right: Timeline of animal experiments and daily H_2_ treatment. **B**. Capture of the gastrocnemius strike area and blood perfusion 0-72 hours post-wounding in the 66% H_2_ Control groups. **C**. & **D**. Quantification of diameter and blood perfusion of the gastrocnemius strike area between two groups. **E**. & **F**. Representative immunofluorescence images for NFH (green) nerve injury repair and axons maturation in dermal wounds of at day 3 and 11 post wounding.

Data in **C** & **D** were processed unpaired T test. All of the data are plotted as Mean±SEM. * *P*-value < 0.05; ** *P*-value <0.01; *** *P*-value < 0.001; no stars for *P*-value > 0.05; Scale bar = 100 μm. White dotted line in **E** & **F** indicates the boundary between the epithelium and dermis. Arrow in **E** & **F** indicates NFH^+^ cells.

**Table. S1. List of primary and secondary antibodies used in experiments.** IHC indicates immunochemistry, IF indicates immunofluorescence; FC indicates flow cytometry.

**A. Primary antibody list**

| Antigen | Host Species | Type | Conjugates | Dillution | Supplier | Reference |
| --- | --- | --- | --- | --- | --- | --- |
| CD31 | Rabbit IgG | polyclonal | unconjugated | 1:50-IF | Abcam | ab28364 |
| VEGFA | Rabbit IgG | monoclonal | unconjugated | 1:500-IF | Abcam | ab52917 |
| CD34 | Rabbit IgG | monoclonal | unconjugated | 1:200-IF | Abcam | ab81289 |
| Cytokeratin 14 | Rabbit IgG | monoclonal | unconjugated | 1:1000-IF | Abcam | ab181595 |
| a-SMA | Rabbit IgG | monoclonal | unconjugated | 1:500-IF | Abcam | ab32575 |
| Collagen I | Rabbit IgG | polyclonal | unconjugated | 1:500-IF,  1:200-IHC | Abcam | Ab254113 |
| Collagen III | Rabbit IgG | polyclonal | unconjugated | 1:1000 IHC | proteintech | 22734-1-AP |
| Ki67 | Rabbit IgG | polyclonal | unconjugated | 1:500-IF,  1:200-IHC | Abcam | ab15580 |
| Cytokeratin 15 | Rabbit IgG | polyclonal | unconjugated | 1:100-IHC | Biodragon | BD-PT1263 |
| P63 | Rabbit IgG | polyclonal | unconjugated | 1:100-IHC | Biodragon | BD-PT3547 |
| Lgr6 | Rabbit IgG | monoclonal | unconjugated | 1:100-IHC | Abcam | ab126747 |
| Fibronectin | Rabbit IgG | polyclonal | unconjugated | 1:200-IHC | Abcam | ab2413 |
| Laminin beta 1 | Rabbit IgG | polyclonal | unconjugated | 1:100-IHC | proteintech | 23498-1-AP |
| Integrin-β1 | Rabbit IgG | polyclonal | unconjugated | 1:200-IHC | proteintech | 12594-1-AP |
| Collagen XVII | Rabbit IgG | monoclonal | unconjugated | 1:100-IHC | proteintech | ab184996 |
| NF-H | Rabbit IgG | polyclonal | unconjugated | 1:200-IF | proteintech | 21471-1-AP |
| CD16/32  (For Blocking) | Rat | monoclonal | unconjugated | 1:50-FC | Biolegend | 101319 |
| CD3 | Rat IgG2b,κ | monoclonal | APC/Cyanine7 | 1:80-FC | Biolegend | 100222 |
| CD4 | Rat IgG2a,κ | monoclonal | Alexa Fluor® 488 | 1:900-FC | Biolegend | 100529 |
| CD8a | Rat IgG2a,κ | monoclonal | Alexa Fluor® 647 | 1:250-FC | Biolegend | 100724 |
| T-bet | Mouse IgG1, κ | monoclonal | PE | 1:20-FC | Biolegend | 644810 |
| IFN-γ | Rat IgG1, κ | monoclonal | Alexa Fluor® 647 | 1:200-FC | Biolegend | 505814 |
| GATA3 | Mouse IgG2b, κ | monoclonal | Alexa Fluor® 647 | 1:25-FC | Biolegend | 653810 |
| IL-4 | Rat IgG1, κ | monoclonal | PE | 1:80-FC | Biolegend | 504104 |
| IL-17A | Rat IgG1, κ | monoclonal | PE | 1:80-FC | Biolegend | 506904 |
| CD25 | Rat IgG2b,κ | monoclonal | PE | 1:20-FC | Biolegend | 101904 |
| CD127 | Rat IgG2a,κ | monoclonal | Brilliant Violet 421™ | 1:20-FC | Biolegend | 135024 |

**B. Secondary antibody list**

| Antigen | Host Species | Type | Conjugates | Dillution | Supplier | Reference |
| --- | --- | --- | --- | --- | --- | --- |
| Rabbit IgG(H+L) , | Goat | F(ab')2  Fragment | Alexa Fluor ® 488 | 1:1000-IF | Cell Signaling | #4412 |
| Rat IgG | Goat | Polyclonal | Alexa Fluor® 594) | 1:1000-IF | Abcam | ab150160 |
| Mouse and Rabbit IgG | Goat | Polyclonal | HRP | 1:1-IHC | ZSGB-BIO | PV-9000 |

**Table S2. Gradient conditions for reversed phase C18 separation**

| Time (min) | A (v%) | B (v%) |
| --- | --- | --- |
| 0 | 100 | 0 |
| 2 | 70 | 30 |
| 9 | 30 | 70 |
| 11 | 5 | 95 |
| 12 | 0 | 100 |
| 14 | 0 | 100 |
| 14.1 | 100 | 0 |
| 16 | 100 | 0 |

**Table S3. Gradient conditions for HILIC separation of polar metabolites**

| Time (min) | A (v%) | B (v%) |
| --- | --- | --- |
| 0 | 95 | 5 |
| 1 | 95 | 5 |
| 7 | 50 | 50 |
| 12 | 50 | 50 |
| 12.1 | 95 | 5 |

**Table S10. Top 20 significant pathways discovered by nontargeted metabolomic analysis between the D3H and D3C groups**

| **Pathway Name** | **-log(p)** | **Impact** |
| --- | --- | --- |
| Aminoacyl-tRNA biosynthesis | 22.02 | 0.11268 |
| Cyanoamino acid metabolism | 9.8804 | 0 |
| **Arginine and proline metabolism** | **7.8377** | **0.30285** |
| Nitrogen metabolism | 7.1529 | 0.0083 |
| Cysteine and methionine metabolism | 6.0914 | 0.05003 |
| **Alanine, aspartate, and glutamate metabolism** | **5.1297** | **0.31054** |
| Valine, leucine, and isoleucine biosynthesis | 4.8976 | 0.0265 |
| Valine, leucine, and isoleucine degradation | 4.1352 | 0.02232 |
| Histidine metabolism | 3.9533 | 0.0056 |
| Glycine, serine, and threonine metabolism | 3.7884 | 0.13604 |
| D-arginine and D-ornithine metabolism | 3.2378 | 0 |
| Sulfur metabolism | 2.4496 | 0 |
| Sphingolipid metabolism | 2.137 | 0 |
| Pantothenate and CoA biosynthesis | 2.0645 | 0 |
| Phenylalanine, tyrosine, and tryptophan biosynthesis | 2.0645 | 0.00062 |
| beta-alanine metabolism | 2.0304 | 0 |
| Lysine biosynthesis | 1.9059 | 0 |
| Methane metabolism | 1.8498 | 0.01751 |
| Glutathione metabolism | 1.7476 | 0 |
| Nicotinate and nicotinamide metabolism | 1.6144 | 0 |

**Legends for Movies S1&S2**

**Movie S1. Live cell imaging of HPF cells under H_2_ medium condition within 24 h.** HPF cells stained by actin-GFP images were taken every one hour, and 0, 4, 8, 12, 16, 20 and 24 h images were collected to build the video. Bar indicates 1000 μ.

**Movie S2. Live cell imaging of HPF cells under normal condition within 24 h.** HPF cells stained by actin-GFP images were taken every one hour, and 0, 4, 8, 12, 16, 20 and 24 h images were collected to build the video. Bar indicates 1000 μM.
